# Supplementary material for: Piperacillin/tazobactam resistance in a clinical isolate of Escherichia coli due to IS26-mediated amplification of blaTEM-1B
Source: Nat Commun. 2020 Oct 1;11:4915. doi: 10.1038/s41467-020-18668-2 (PMC7530762; doi:10.1038/s41467-020-18668-2)
Supplement: Supplementary file 1 — Supplementary Information [file 41467_2020_18668_MOESM1_ESM.pdf]

Piperacillin/tazobactam resistance in a clinical isolate of *Escherichia coli* due to IS26-mediated amplification of *bla*$_{\text{TEM-1B}}$.

Hubbard *et al*.

Supplementary Methods 1:

DNA extraction

Genomic DNA for qPCR was extracted using the PureGene® Yeast/Bact kit B (Qiagen, Germany) following the manufacturer's instructions for extraction of DNA from Gram-negative bacteria after normalising each culture to an optical density at 600nm (OD_{600}) of 1 and eluted in molecular grade water.

Long fragment genomic DNA extraction for PCR amplification and both Illumina and Oxford Nanopore Technologies (ONT) sequencing was performed using the Genomic DNA Buffer Set, following the manufacturer's instructions for genomic DNA extraction using the Genomic-tip 100/G (both Qiagen, Germany) but eluted in molecular grade water.

Plasmid extraction was performed from 100 ml LB broth (LB) cultures with the Plasmid Midi Kit using Genomic-Tip 100 (both Qiagen, Germany) following the manufacturer's instructions and eluted in molecular grade water.

Total DNA concentration was measured using a QuBit 4.0 Fluorometer (Invitrogen, US), with the dsDNA Broad Range kit.

PCR

A single replicate of all PCR reactions was performed using 0.02 U/µl Q5® High-Fidelity DNA Polymerase, 1x Q5® Reaction Buffer, 200 µM dNTPs and 0.5 µM of each primer (Supplementary Table 4) in a total volume of 50 µl.

16S rRNA PCR amplification was performed using the following protocol: denaturation at 98°C for 30 seconds, followed by 45 cycles of denaturation at 98°C for 10 seconds, annealing at 53°C for 30 seconds and elongation at 72°C for 30 seconds, followed by a single, final extension of 2 minutes at 72°C.

The detection of the pseudo-compound transposon or translocatable unit (TU) was performed using the following PCR protocol: denaturation at 98°C for 30 seconds, followed by 35 cycles of denaturation at 98°C for 10 seconds, annealing at 68°C (left junction of the pseudo-compound transposon in the chromosome and the tandem array of TUs) or 67°C (right junction of the pseudo-compound transposon in the chromosome) for 30 seconds and elongation at 72°C for 17 seconds (left junction of the pseudo-compound transposon in the chromosome), 25 seconds (right junction of the pseudo-compound transposon in the chromosome) or 20 seconds (tandem array of TUs), followed by a single, final extension of 2 minutes at 72°C.

Detection of the formation of junctions in the pHSG396:IS*26* due to the insertion of the TU using the following PCR protocol: denaturation at 98°C for 30 seconds, followed by 35 cycles of denaturation at 98°C for 10 seconds, annealing at 68°C (left junction of the insertion) or 67°C (right junction of the insertion) for 30 seconds and elongation at 72°C for 15 seconds (left junction) and 14 seconds (right junction), followed by a single, final extension of 2 minutes at 72°C.

The PCR products from the left side of the pseudo-compound transposon in the chromosome and the TU were PCR purified using the Monarch® PCR and DNA Clean-up Kit (New England Biolabs (NEB), USA) following the manufacturer's protocol and eluted in molecular grade water. The PCR products from the right side of the pseudo-compound transposon in the chromosome and the left and right junction of the TU insertion into pHSG396:IS*26* were extracted from the gel using the Monarch® DNA Gel Extraction Kit (NEB, USA) following the manufacturer's instructions and eluted in molecular grade water. All purified DNA extracts were then Sanger sequenced by GeneWiz (Takley, UK).

*IS*26 *plasmid construct*

IS*26* from 190963 (TZP-susceptible isolate) was amplified using 0.02 U/µl Q5® High-Fidelity DNA Polymerase, 1x Q5® Reaction Buffer, 200 µM dNTPs and 0.5 µM of the primers CMCap_F and CMCap_R (Supplementary Table 4) in a total volume of 50 µl. Amplification was performed using the

following protocol; Denaturation at 98°C for 30 seconds, followed by 35 cycles of denaturation at 98°C for 10 seconds, annealing at 69°C for 30 seconds and elongation at 72°C for 9 seconds, followed by a single, final extension step 72°C for 2 minutes.

The restriction sites for the restriction enzymes EcoRI and KpnI were added to the 5' ends of the 820 bp double stranded amplicon by a second round of PCR, using the primers CMCapRE_F and CMCapRE_R (Supplementary Table 4). Amplification was performed using the same protocol as the initial amplification of IS*26*, except with an annealing temperature of 72°C for 30 seconds.

The resulting PCR product and the recipient plasmid pHSG396 were digested with the restriction enzymes EcoRI and KpnI (both NEB, US). PCR clean-up was then performed using the Monarch PCR and DNA Clean-up Kit following the manufacturer's instructions. The digested IS*26* PCR product and pHSG396 were ligated together using T4 ligase (NEB, US), at a 3:1 insert to vector mass ratio, incubated at room temperature for 10 minutes.

The ligated plasmid (5 µl) was transformed into NEB® 5-alpha competent *E. coli* (NEB, US) using the following protocol; the reaction was incubated on ice for 2 minutes, then heat shocked at 42°C for 30 seconds. Following a second incubation on ice for 2 minutes, 950 µL of SOC outgrowth medium was added and incubated at 37°C, 250 rpm for 1 hour. The transformed cells were plated out onto LB agar supplement with 35 µg/ml chloramphenicol and Isopropyl β-D-1-thiogalactopyranoside/X-gal (Fisher Scientific, USA) and incubated at 37°C for 18 hours and the plasmid was extracted following the protocol above. IS*26* in pHSG396 was Sanger sequenced by GeneWiz (Takely, UK) using commercially available M13 primers.

Supplementary Table 1: Resistance profile of paired isolates determined in clinic. Resistance profile of cefpodoxime (CPD), cefoxitin (FOX), piperacillin/tazobactam (TZP), meropenem (MEM), ciprofloxacin (CIP), cefotetan (CTT), amikacin (AMK), ertapenem (ETP), amoxicillin/clavulanic acid (AMC), chloramphenicol (CHL) and ampicillin (AMP) towards identified paired isolates with presumptive clonality assessed in clinic by disk diffusion method. Disk diffusion antimicrobial susceptibility testing was performed as a single replicate (*n*=1).

Patient	Isolate Number	CPD	FOX	TZP	MEM	CIP	CTT	AMK	ETP	AMC	CHL	AMP
1	101737	S	S	S	S	S	S	S	S	S	-	-
	102167	S	S	R	S	R	S	S	S	I	-	-
2	153964	S	S	S	S	S	S	S	S	R	S	R
	152025	S	S	R	S	S	S	S	S	R	S	R
3	190693	S	S	S	S	R	R	S	S	R	S	R
	169757	S	S	R	S	R	R	S	S	R	S	R

Supplementary Table 2: TEM-1B is likely to be the most important determinant of piperacillin/tazobactam resistance. Minimum inhibitory concentrations (MIC) of piperacillin supplemented with 4 µg/ml tazobactam, 8 µg/ml tazobactam, 16 µg/ml tazobactam and 4 µg/ml tazobactam plus 100 mM sodium chloride. Each MIC were performed in triplicate (*n*=3).

	Piperacillin plus 4 µg/ml tazobactam	Piperacillin plus 8 µg/ml tazobactam	Piperacillin plus 16 µg/ml tazobactam	Piperacillin plus 4 µg/ml tazobactam and 100 mM NaCl
TZP-resistant	128 µg/ml	64 µg/ml	16 µg/ml	64 µg/ml

Supplementary Table 3: Mean and standard error of the mean of all data presented in this study.

Assay and figure	Isolate	Gene	Media	Mean	Standard Error of the mean (±)
Nitrocefin assay					
Fig. 1A	TZP-susceptible	-	-	0.041	0.002
Fig. 1A	TZP-resistant	-	-	0.208	0.011
qPCR					
Fig. 1B	TZP-susceptible	aac(3)-lla	-	1.089	0.037
		aac(6')-lb-cr	-	1.226	0.068
		bla_{OXA-1}	-	1.516	0.02
		bla_{TEM-1B}	-	1.207	0.047
		tet(D)	-	0.525	0.016
Fig. 1B	TZP-resistant	aac(3)-lla	-	8.523	0.105
		aac(6')-lb-cr	-	8.2	0.139
		bla_{OXA-1}	-	7.252	0.375
		bla_{TEM-1B}	-	9.484	0.354
		tet(D)	-	9.154	0.286

Fig. 3	TZP-susceptible - no TZP	aac(3)-IIa	-	0.734	0.062
		aac(6')-Ib-cr	-	0.875	0.005
		bla_{OXA-1}	-	1.199	0.041
		bla_{TEM-1B}	-	1.002	0.063
		tet(D)	-	1.292	0.104
Fig. 3	TZP-susceptible - TZP	aac(3)-IIa	-	3.104	0.339
		aac(6')-Ib-cr	-	3.249	0.377
		bla_{OXA-1}	-	3.708	0.685
		bla_{TEM-1B}	-	3.604	0.811
		tet(D)	-	5.641	0.785
Fig. 3	TZP-susceptible + IS26 - TZP	aac(3)-IIa	-	6.37	0.468
		aac(6')-Ib-cr	-	7.379	0.355
		bla_{OXA-1}	-	8.571	0.369
		bla_{TEM-1B}	-	10.068	0.896
		tet(D)	-	10.919	1.153

Fig. 3	TZP-susceptible + IS26 - CHL only	aac(3)-lla	-	-0.627	0.297
		aac(6')-lb-cr	-	1.128	0.186
		bla_{OXA-1}	-	0.8	0.283
		bla_{TEM-1B}	-	1.257	0.105
		tet(D)	-	0.087	1.284
Comparative fitness					
Fig. 4	TZP-susceptible TZP evolved	-	ISO	0.891	0.051
		-	LB	1.105	0.062
		-	M9	1.068	0.033
Fig. 4	TZP-resistant	-	ISO	0.894	0.055
		-	LB	1.051	0.031
		-	M9	0.933	0.14

Supplementary Table 4: Primer name and sequences used for PCR amplification and qPCR in this study.

Primer name	Primer Sequence
16S-27F	AGAGTTTGATCCTGGCTCAG
16S-1492R	TACCTTGTTACGACTT
RM2_F1	TCTTCCCACTGCTGACGAAC
RM2_R1	AGTGTGACGGAATCGTTGCT
RM2_F2	CGCCCGAGATACAACATCCA
RM2_R2	GTTGCTTTTTCTCGACGTGCT
CMCap_F	GGCACTGTTGCAAATAGTCGGT
CMCap_R	GGCACTGTTGCAAAGTTAGCGA
CMCapRE_F	AAAAAGAATTCGGCACTGTTGCAAATAGTCGGT
CMCapRE_R	AAAAAGGTACCGGCACTGTTGCAAAGTTAGCGA
CapCon_F1	GGGGAAACGCCTGGTATCTT
CapCon_R1	TGACGGAATCGTTGCTGTTG
CapCon_F2	GCTGGGAATAGAACAGCCGA
CapCon_R2	CGGCTCGTATGTTGTGTGGA
uidA_F	TCTGGCAACCGGGTGAAG
uidA R	TAGATATCACACTCTGTCTGGCT
TEM_F	GGAACCGGAGCTGAATGAAG
TEM_R	TCAGCAATAAACCAGCCAGC

OXA-1_F	TGCATCCACAAACGCTGAAA
OXA-1-_R	TGCGAAACCCAAACAACAGA
tetD_F	AGCAGAAACAAGAAAGCGCA
tetD_R	TTCTCACTCAGCCGTTTTGC
aac(3)-lla_F	GCATGCCTCACTTAAAGCGA
aac(3)-lla_R	ATTGATTCAGCAGGCCGAAC
aac(6')-lb-cr_F	CCCAGTCGTACGTTGCTCTT
aac(6')-lb-cr_F	CCTCGGGATCATTGAACAGC

A

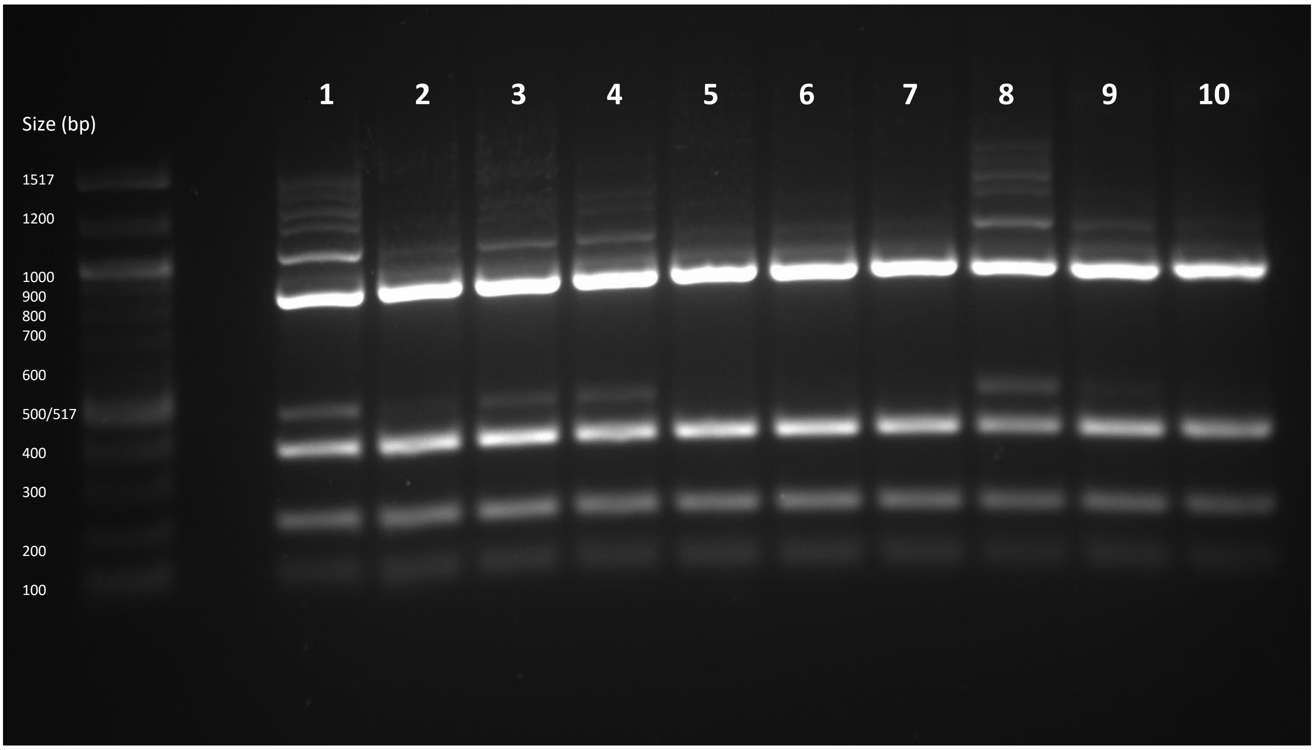
1
2
3
4
5
6
7
8
9
10
Size (bp)
1517
1200
1000
900
800
700
600
500/517
400
300
200
100

B

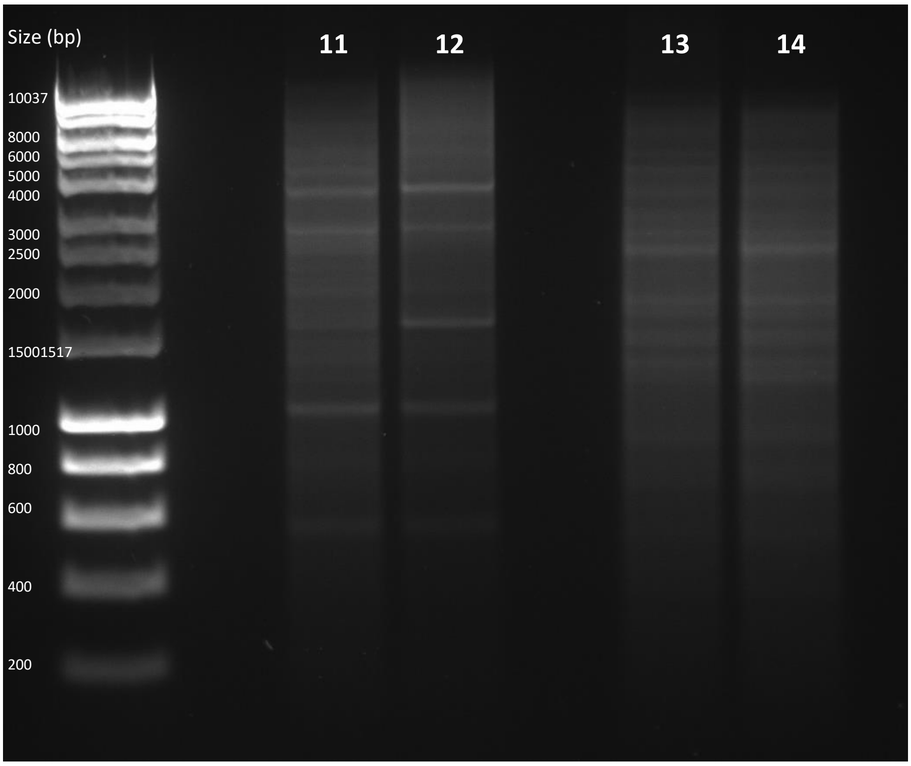
Size (bp)
11
12
13
14
10037
8000
6000
5000
4000
3000
2500
2000
15001517
1000
800
600
400
200

Supplementary Figure 1: Only one pair isolates are presumptive clones. Restriction fragment length polymorphism (RFLP) of **A**) 16S rRNA PCR amplicon of **1**) 151985, **2**) 153006, **3**) 101737, **4**) 102167, **5**) 153964, **6**) 152025, **7**) 155512, **8**) 154810, **9**) 190693 and **10**) 169757 digested with AlwNI, PpuHI and MslI. RFLP of **B**) long fragment genomic DNA extraction from **11**) 153964, **12**) 152025, **13**) 190693 and **14**) 169757 digested with SpeI and MslI. RFLP was performed as a single replicate (*n*=1). Source data are provided as a Source Data file.

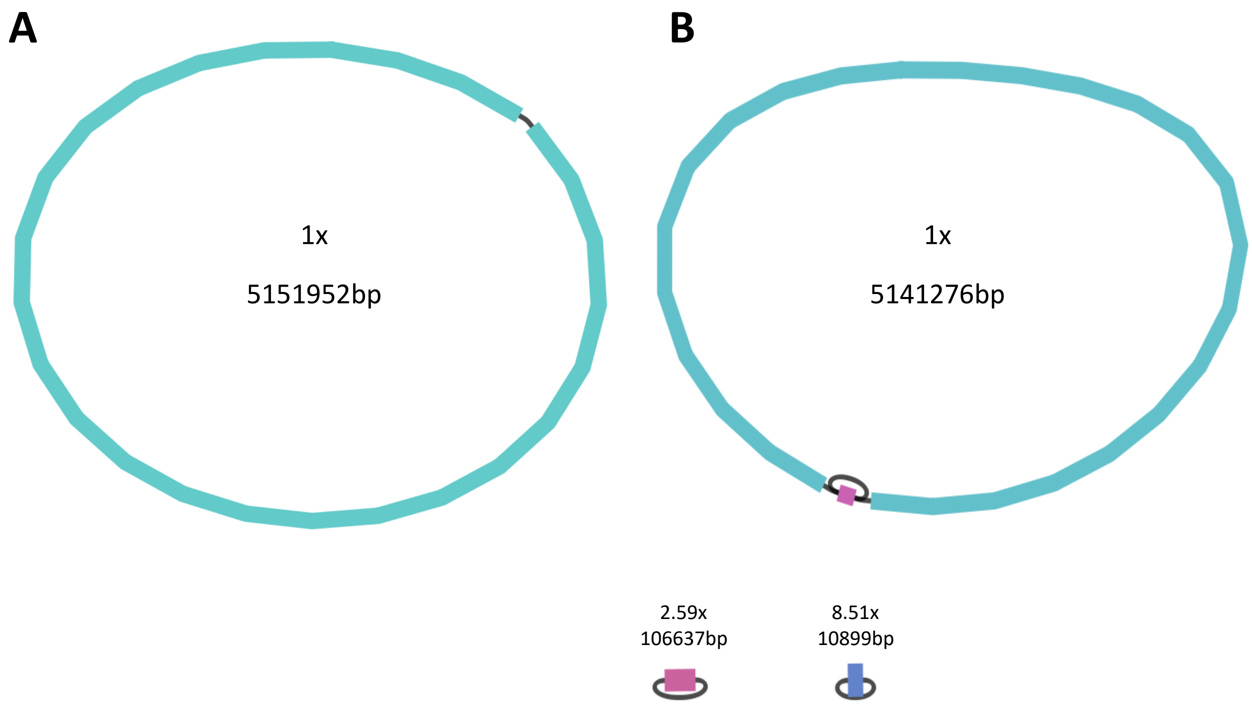

Supplementary Figure 2: The piperacillin/tazobactam-resistant isolate contained a plasmid and circular DNA molecule which were not present in the piperacillin/tazobactam-susceptible isolate. Visualisation of the hybrid assembly of long and short read sequencing reads by Unicycler of the **A**) piperacillin/tazobactam (TZP)-susceptible isolate and **B**) and TZP-resistant isolate.

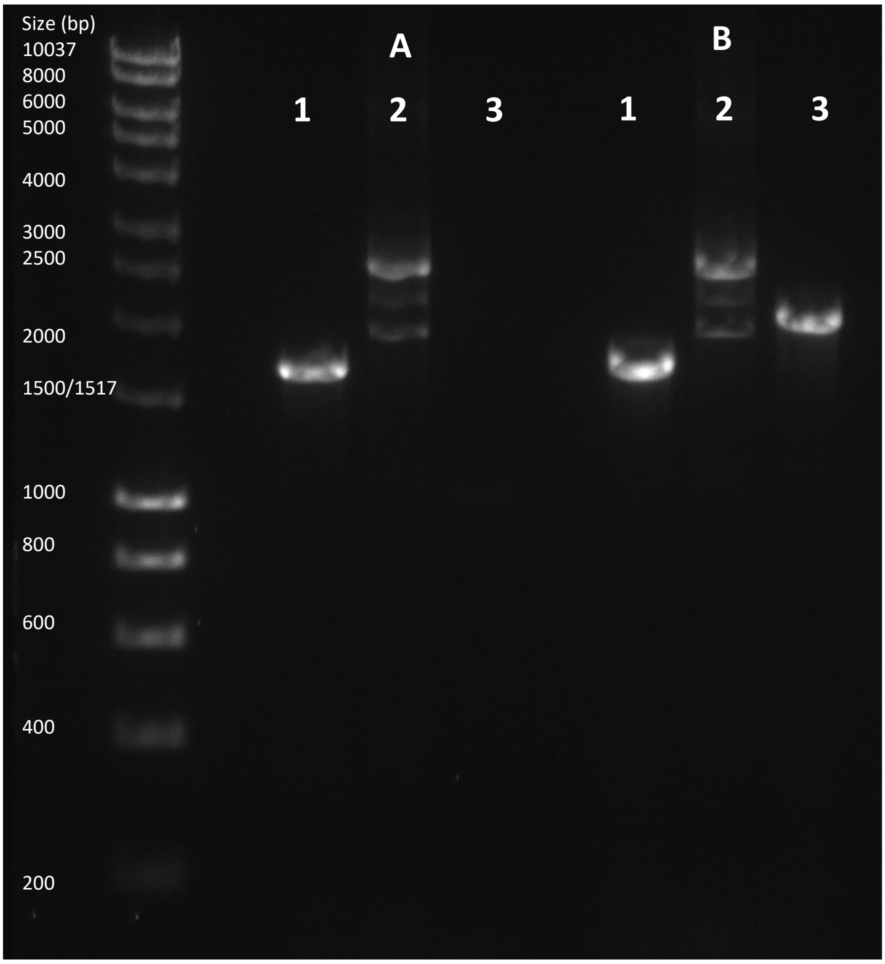

Supplementary Figure 3: PTn*6762* is present in the chromosome of both the piperacillin/tazobactam-resistant and piperacillin/tazobactam-susceptible isolate but exists as a tandem array in the resistant isolate only. Gel electrophoresis of the PCR amplicons of the **1**) left junction of the chromosomally located IS*26* and resistance module, **2**) right junction of the chromosomally located IS*26* and resistance module and **3**) translocatable unit (TU) tandem array from **A**) piperacillin/tazobactam (TZP)-susceptible isolate and **B**) TZP-resistant isolate**.** PCR amplification was performed as a single replicate (*n*=1). Source data are provided as a Source Data file.

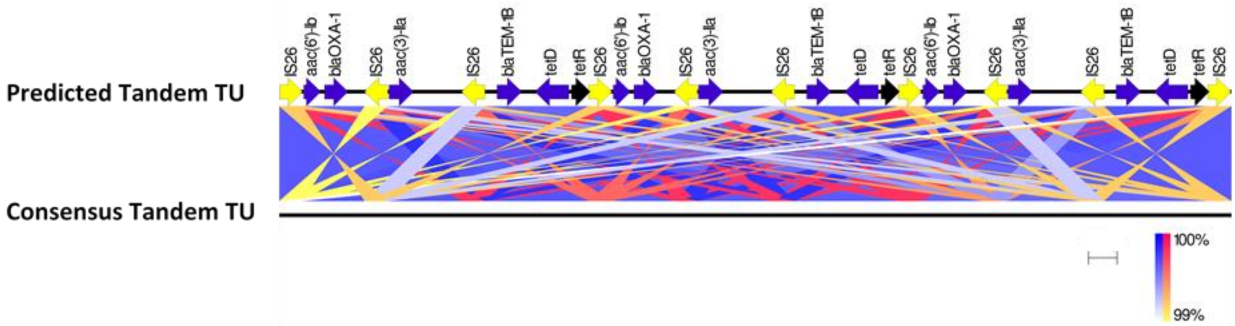

Supplementary Figure 4: The translocate unit exists as a tandem array in the chromosome of the piperacillin/tazobactam resistant isolate. Comparison of the nucleotide sequence of the predicted structure of three tandem translocatable units (TU) to the consensus sequence built from mapped Oxford Nanopore Technologies sequencing reads. Yellow arrows represent IS*26*, blue arrows represent antimicrobial resistance genes and black arrows represent *tetR*. Percentage relatedness between the predicted and consensus sequences in the same orientation are represented in by the blue to white scale and percentage relatedness between the predicted and consensus sequences in the opposite direction are represented in the by the pink to yellow scale. Scale equals 1000 base pairs.

A)

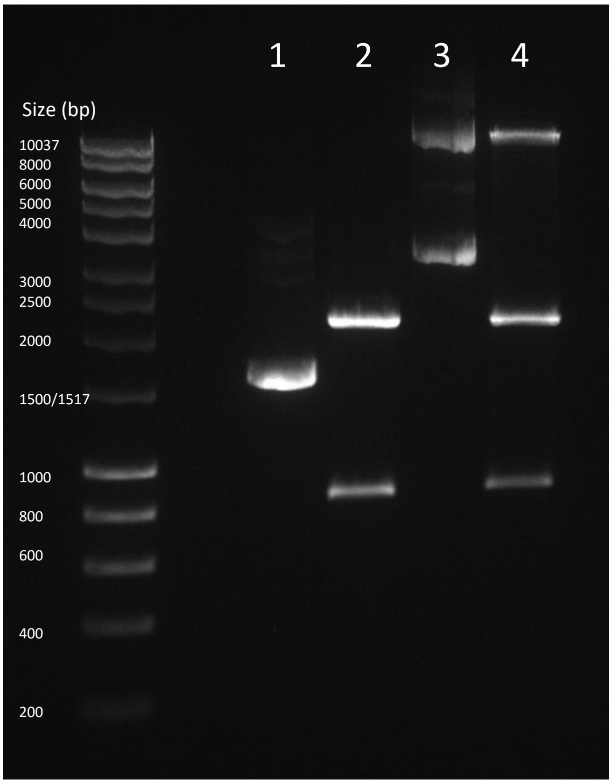
1
2
3
4
Size (bp)
10037
8000
6000
5000
4000
3000
2500
2000
1500/1517
1000
800
600
400
200

B)

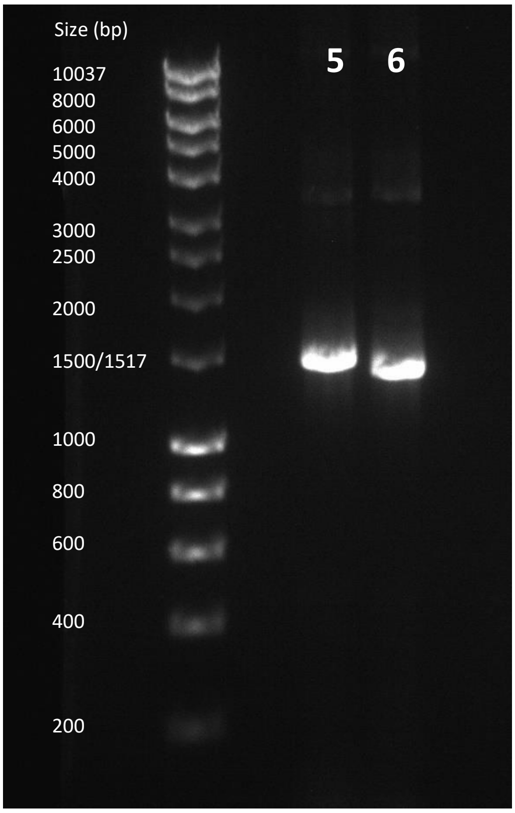
Size (bp)
5
6
10037
8000
6000
5000
4000
3000
2500
2000
1500/1517
1000
800
600
400
200

Supplementary Figure 5: The translocatable unit was captured in the pHSG396:IS*26* plasmid following replication of the evolutionary event. A) Gel electrophoresis of **1**) undigested pHSG396:IS*26*, **2**) pHSG396:IS*26* digested with XhoI and EcoRI, **3**) undigested pHSG396:IS26 after growth in the presence of 8/4 µg/ml piperacillin/tazobactam (TZP) and transformation in New England Biolabs (NEB)® 5-alpha competent *Escherichia coli* and **4**) pHSG396:IS26 digested with XhoI and EcoRI after growth in the presence of 8/4 µg/ml TZP and transformation in NEB® 5-alpha competent *E. coli* and **B**) gel electrophoresis of PCR amplicons from the **5**) left and **6**) right junctions of pHSG396:IS*26* following insertion of the translocatable unit. Restriction digest and PCR amplification were performed as a single replicate (*n*=1). Source data are provided as a Source Data file.

A

TCGACGGAGTTCCACTGAGCGTCAGACCCCGTAGAAAAGATCAAAGGATCTTCTTGAGATCCTTTTTTTCTGCGC

GTAATCTGCTGCTTGCAAACAAAAAAACCACCGCTACCAGCGGTGGTTTGTTTGCCGGATCAAGAGCTACCAA

CTCTTTTTCCGAAGGTAACTGGCTTCAGCAGAGCGCAGATACCAAATACTGTTCTTCTAGTGTAGCCGTAGTTA

GGCCACCACTTCAAGAACTCTGTAGCACCGCCTACATACCTCGCTCTGCTAATCCTGTTACCAGTGGCTGCTGC

CAGTGGCGATAAGTCGTGTCTTACCGGGTTGGACTCAAGACGATAGTTACCGGATAAGGCGCAGCGGTCGGG

CTGAACGGGGGGTTCGTGCACACAGCCCAGCTTGGAGCGAACGACCTACACCGAACTGAGATACCTACAGCG

TGAGCTTATGAGAAAGCGCCACGCTTCCCGAAGGGAGAAAGGCGGACAGGTATCCGGTAAGCGGCAGGGTC

GGAACAGGAGAGCGCACGAGGGAGCTTCCAGGGGGAAACGCCTGGTATCTTTATAGTCCTGTCGGGTTTCGC

CACCTCTGACTTGAGCGTCGATTTTTGTGATGCTCGTCAGGGGGGCGGAGCCTATGGAAAAACGCCAGCAAC

GCGGCCTTTTTACGGTTCCTGGCCTTTTGCTGGCCTTTTGCTCACATGTTCTTTCCTGCGTTATCCCCTGATTCTG

TGGATAACCGTATTACCGCCTTTGAGTGAGCTGATACCGCTCGCCGCAGCCGAACGACCGAGCGCAGCGAGT

CAGTGAGCGAGGAAGCGGAAGAAGCTCATTCGCCATTCAGGCTGCGCAACTGTTGGGAAGGGCGATCGGTG

CGGGCCTCTTCGCTATTACGCCAGCTGGCGAAAGGGGGATGTGCTGCAAGGCGATTAAGTTGGGTAACGCCA

GGGTTTTCCCAGTCACGACGTTGTAAAACGACGGCCAGTGAATTCGGCACTGTTGCAAATAGTCGGTGGTGAT

AAACTTATCATCCCCTTTTGCTGATGGAGCTGCACATGAACCCATTCAAAGGCCGGCATTTTCAGCGTGACATC

ATTCTGTGGGCCGTACGCTGGTACTGCAAATACGGCATCAGTTACCGTGAGCTGCAGGAGATGCTGGCTGAA

CGCGGAGTGAATGTCGATCACTCCACGATTTACCGCTGGGTTCAGCGTTATGCGCCTGAAATGGAAAAACGG

CTGCGCTGGTACTGGCGTAACCCTTCCGATCTTTGCCCGTGGCACATGGATGAAACCTACGTGAAGGTCAATG

GCCGCTGGGCGTATCTGTACCGGGCCGTCGACAGCCGGGGCCGCACTGTCGATTTTTATCTCTCCTCCCGTCG

TAACAGCAAAGCTGCATACCGGTTTCTGGGTAAAATCCTCAACAACGTGAAGAAGTGGCAGATCCCGCGATTC

ATCAACACGGATAAAGCGCCCGCCTATGGTCGCGCGCTTGCTCTGCTCAAACGCGAAGGCCGGTGCCCGTCTG

ACGTTGAACACCGACAGATTAAGTACCGGAACAACGTGATTGAATGCGATCATGGCAAACTGAAACGGATAA

TCGGCGCCACGCTGGGATTTAAATCCATGAAGACGGCTTACGCCACCATCAAAGGTATTGAGGTGATGCGTG

CACTACGCAAAGGCCAGGCCTCAGCATTTTATTATGGTGATCCCCTGGGCGAAATGCGCCTGGTAAGCAGAGT

TTTTGAAATGTAAGGCCTTTGAATAAGACAAAAGGCTGCCTCATCGCTAACTTTGCAACAGTGCCGGTACCCC

ATCGATGGGGGATCCTCTAGAGTCGACCCCTCGAGGGGCATGCAAGCTTGGCGTAATCATGGTCATAGCTGTT

TCCTGTGTGAAATTGTTATCCGCTCACAATTCCACACAACATACGAGCCGGAAGCATAAAGTGTAAAGCCTGG

GGTGCCTAATGAGTGAGCTAACTCACATTAATTGCGTTGCGCTCACTGCCCGCTTTCCAGTCGGGAAACCTGTC

GTGCCAGCTGCATTAATGAATCGGCCAACGCGCGGGGAGAGGCGGTTTGCGTATTGAGCTTGGCACTGGGCC

AAGCTGAATTTCTGCCATTCATCCGCTTATTATCACTTATTCAGGCGTAGCAACCAGGCGTTTAAGGGCACCAA

TAACTGCCTTAAAAAAATTACGCCCCGCCCTGCCACTCATCGCAGTACTGTTGTAATTCATTAAGCATTCTGCCGA

CATGGAAGCCATCACAAACGGCATGATGAACCTGAATCGCCAGCGGCATCAGCACCTTGTCGCCTTGCGTATA

ATATTTGCCCATGGTGAAAACGGGGGCGAAGAAGTTGTCCATATTGGCCACGTTTAAATCAAAACTGGTGAA

ACTCACCCAGGGATTGGCTGAGACGAAAAACATATTCTCAATAAACCCTTTAGGGAAATAGGCCAGGTTTTCA

CCGTAACACGCCACATCTTGCGAATATATGTGTAGAAACTGCCGGAAATCGTCGTGGTATTCACTCCAGAGCG

ATGAAAACGTTTCAGTTTGCTCATGGAAAACGGTGTAACAAGGGTGAACACTATCCCATATCACCAGCTCACC

GTCTTTCATTGCCATACGAAATTCCGGATGAGCATTCATCAGGCGGGCAAGAATGTGAATAAAGGCCGGATA

AAACTTGTGCTTATTTTTCTTTACGGTCTTTAAAAAGGCCGTAATATCCAGCTGAACGGTCTGGTTATAGGTAC

ATTGAGCAACTGACTGAAATGCCTCAAAATGTTCTTTACGATGCCATTGGGATATATCAACGGTGGTATATCCA

GTGATTTTTTTCTCCATTTTAGCTTCCTTAGCTCCTGAAAATCTCGATAACTCAAAAAATACGCCCGGTAGTGAT

CTTATTTCATTATGGTGAAAGTTGGAACCTCTTACGTGCCGATCAACGTCTCATTTTCGCCAAAAGTTGGCCCA

GGGCTTCCCGGTATCAACAGGGACACCAGGATTTATTTATTCTGCGAAGTGATCTTCCGT

B

GGCACTGTTGCAAATAGTCGGTGGTGATAAACTTATCATCCCCTTTTGCTGATGGAGCTGCACATGAACCCATT

CAAAGGCCGGCATTTTCAGCGTGACATCATTCTGTGGGCCGTACGCTGGTACTGCAAATACGGCATCAGTTAC

CGTGAGCTGCAGGAGATGCTGGCTGAACGCGGAGTGAATGTCGATCACTCCACGATTTACCGCTGGGTTCAG

CGTTATGCGCCTGAAATGGAAAAACGGCTGCGCTGGTACTGGCGTAACCCTTCCGATCTTTGCCCGTGGCACA

TGGATGAAACCTACGTGAAGGTCAATGGCCGCTGGGCGTATCTGTACCGGGCCGTCGACAGCCGGGGCCGCA

CTGTCGATTTTTATCTCTCCTCCCGTCGTAACAGCAAAGCTGCATACCGGTTTCTGGGTAAAATCCTCAACAAC

GTGAAGAAGTGGCAGATCCCGCGATTCATCAACACGGATAAAGCGCCCGCCTATGGTCGCGCGCTTGCTCTG

CTCAAACGCGAAGGCCGGTGCCCGTCTGACGTTGAACACCGACAGATTAAGTACCGGAACAACGTGATTGAA

TGCGATCATGGCAAACTGAAACGGATAATCGGCGCCACGCTGGGATTTAAATCCATGAAGACGGCTTACGCC

ACCATCAAAGGTATTGAGGTGATGCGTGCACTACGCAAAGGCCAGGCCTCAGCATTTTATTATGGTGATCCCC

TGGGCGAAATGCGCCTGGTAAGCAGAGTTTTTGAAATGTAAGGCCTTTGAATAAGACAAAAGGCTGCCTCAT

CGCTAACTTTGCAACAGTGCCTTTAAGCGTGCATAATAAGCCCTACACAAATTGGGAGTTAGACATCATGAGC

AACGCAAAAACAAAGTTAGGCATCACAAAGTACAGCATCGTGACCAACAGCAACGATTCCGTCACACTGCGCC

TCATGACTGAGCATGACCTTGCGATGCTCTATGAGTGGCTAAATCGATCTCATATCGTCGAGTGGTGGGGCGG

AGAAGAAGCACGCCCGACACTTGCTGACGTACAGGAACAGTACTTGCCAAGCGTTTTAGCGCAAGAGTCCGT

CACTCCATACATTGCAATGCTGAATGGAGAGCCGATTGGGTATGCCCAGTCGTACGTTGCTCTTGGAAGCGGG

GACGGACGGTGGGAAGAAGAAACCGATCCAGGAGTACGCGGAATAGACCAGTTACTGGCGAATGCATCACA

ACTGGGCAAAGGCTTGGGAACCAAGCTGGTTCGAGCTCTGGTTGAGTTGCTGTTCAATGATCCCGAGGTCACC

AAGATCCAAACGGACCCGTCGCCGAGCAACTTGCGAGCGATCCGATGCTACGAGAAAGCGGGGTTTGAGAG

GCAAGGTACCGTAACCACCCCATATGGTCCAGCCGTGTACATGGTTCAAACACGCCAGGCATTCGAGCGAACA

CGCAGTGATGCCTAACCCTTCCATCGAGGGGGACGTCCAAGGGCTGGCGCCCTTGGCCGCCCCTCATGTCAAA

CGTTGGGCGAACCCGGAGCCTCATTAATTGTTAGCCGTTAAAATTAAGCCCTTTACCAAACCAATACTTATTAT

GAAAAACACAATACATATCAACTTCGCTATTTTTTTAATAATTGCAAATATTATCTACAGCAGCGCCAGTGCATC

AACAGATATCTCTACTGTTGCATCTCCATTATTTGAAGGAACTGAAGGTTGTTTTTTACTTTACGATGCATCCAC

AAACGCTGAAATTGCTCAATTCAATAAAGCAAAGTGTGCAACGCAAATGGCACCAGATTCAACTTTCAAGATC

GCATTATCACTTATGGCATTTGATGCGGAAATAATAGATCAGAAAACCATATTCAAATGGGATAAAACCCCCA

AAGGAATGGAGATCTGGAACAGCAATCATACACCAAAGACGTGGATGCAATTTTCTGTTGTTTGGGTTTCGCA

AGAAATAACCCAAAAAATTGGATTAAATAAAATCAAGAATTATCTCAAAGATTTTGATTATGGAAATCAAGAC

TTCTCTGGAGATAAAGAAAGAAACAACGGATTAACAGAAGCATGGCTCGAAAGTAGCTTAAAAATTTCACCA

GAAGAACAAATTCAATTCCTGCGTAAAATTATTAATCACAATCTCCCAGTTAAAAACTCAGCCATAGAAAACAC

CATAGAGAACATGTATCTACAAGATCTGGATAATAGTACAAAACTGTATGGGAAAACTGGTGCAGGATTCACA

GCAAATAGAACCTTACAAAACGGATGGTTTGAAGGGTTTATTATAAGCAAATCAGGACATAAATATGTTTTTG

TGTCCGCACTTACAGGAAACTTGGGGTCGAATTTAACATCAAGCATAAAAGCCAAGAAAAATGCGATCACCAT

TCTAAACACACTAAATTTATAAAAAATCTAATGGCAAAATCGCCCAACCCTTCAATCAAGTCGGGACGGCCAA

AAGCAAGCTTTTGGCTCCCCTCGCTGGCGCTCGGCGCCCCTTATTTCAAACGTTAGACGGCAAAGTCACAGAC

CGCGGGATCTCTTATGACCAACTACTTTGATAGCCCCTTCAAAGGCAAGCTGCTTTCTGAGCAAGTGAAGAAC

CCCAATATCAAAGTTGGGCGGTACAGCTATTACTCTGGCTACTATCATGGGCACTCATTCGATGACTGCGCAC

GGTATCTGTTTCCGGACCGTGATGACGTTGATAAGTTGATCATCGGTAGTTTCTGCTCTATCGGGAGTGGGGC

TTCCTTTATCATGGCTGGCAATCAGGGGCATCGGTACGACTGGGCATCATCTTTCCCGTTCTTTTATATGCAGG

AAGAACCTGCATTCTCAAGCGCACTCGATGCCTTCCAAAAAGCAGGTAATACTGTCATTGGCAATGACGTTTG

GATCGGCTCTGAGGCAATGGTCATGCCCGGAATCAAGATCGGGCACGGTGCGGTGATAGGCAGCCGCTCGTT

GGTGACAAAAGATGTGGGGCACTGTTGCAAAGTTAGCGATGAGGCAGCCTTTTGTCTTATTCAAAGGCCTTAC

ATTTCAAAAACTCTGCTTACCAGGCGCATTTCGCCCAGGGGATCACCATAATAAAATGCTGAGGCCTGGCCTTT

GCGTAGTGCACGCATCACCTCAATACCTTTGATGGTGGCGTAAGCCGTCTTCATGGATTTAAATCCCAGCGTG

GCGCCGATTATCCGTTTCAGTTTGCCATGATCGCATTCAATCACGTTGTTCCGGTACTTAATCTGTCGGTGTTCA

ACGTCAGACGGGCACCGGCCTTCGCGTTTGAGCAGAGCAAGCGCGCGACCATAGGCGGGCGCTTTATCCGTG

TTGATGAATCGCGGGATCTGCCACTTCTTCACGTTGTTGAGGATTTTACCCAGAAACCGGTATGCAGCTTTGCT

GTTACGACGGGAGGAGAGATAAAAATCGACAGTGCGGCCCCGGCTGTCGACGGCCCGGTACAGATACGCCC

AGCGGCCATTGACCTTCACGTAGGTTTCATCCATGTGCCACGGGCAAAGATCGGAAGGGTTACGCCAGTACCA

GCGCAGCCGTTTTTCCATTTCAGGCGCATAACGCTGAACCCAGCGGTAAATCGTGGAGTGATCGACATTCACT

CCGCGTTCAGCCAGCATCTCCTGCAGCTCACGGTAACTGATGCCGTATTTGCAGTACCAGCGTACGGCCCACA

GAATGATGTCACGCTGAAAATGCCGGCCTTTGAATGGGTTCATGTGCAGCTCCATCAGCAAAAGGGGATGAT

AAGTTTATCACCACCGACTATTTGCAACAGTGCCTCTCGATAGGTATAGTGTTTTGCAGTTTAGAGGAGATATC

GCGATGCATACGCGGAAGGCAATAACGGAGGCAATTCGAAAATTAGGAGTCCAAACCGGTGACCTGTTGATG

GTGCATGCCTCACTTAAAGCGATTGGTCCGGTCGAAGGAGGAGCGGAGACGGTCGTTGCCGCGTTACGCTCC

GCGGTTGGGCCGACTGGCACTGTGATGGGATACGCGTCGTGGGACCGATCACCCTACGAGGAGACTCTGAAT

GGCGCTCGGTTGGATGACAAAGCCCGCCGTACCTGGCCGCCGTTCGATCCCGCAACGGCCGGGACTTACCGT

GGGTTCGGCCTGCTGAATCAATTTCTGGTTCAAGCCCCCGGCGCGCGGCGCAGCGCGCACCCCGATGCATCG

ATGGTCGCGGTTGGTCCGCTAGCTGAAACGCTGACGGAGCCTCACGAACTCGGTCACGCCTTGGGGGAAGGG

TCGCCCGTCGAGCGGTTCGTCCGCCTTGGCGGGAAGGCCCTGCTGTTGGGTGCGCCGCTAAACTCCGTTACCG

CATTGCACTACGCCGAGGCGGTTGCGGATATCCCCAACAAACGATGGGTGACGTATGAGATGCCGATGCTTG

GAAGAAACGGTGAAGTCGCCTGGAAAACGGCATCAGAATACGATTCAAACGGCATTCTCGATTGCTTTGCTAT

CGAAGGAAAGCCGGATGCGGTCGAAACTATAGCAAATGCTTACGTGAAGCTCGGTCGCCATCGAGAAGGTGT

CGTGGGCTTTGCTCAGTGCTACCTGTTCGACGCGCAGGACATCGTGACGTTCGGCGTCACCTATCTTGAGAAG

CACTTCGGAGCCACTCCGATCGTGCCAGCACACGAAGCCGCCCAGCGCTCTTGCGAGCCTTCCGGTTAGAGGC

CGTCGACAATGATAATCTGGATCAACGGACCTTTCGGCGCCGGAAAGACGACTCTCGCTGAGCGGCTGCGCG

ATCGGCGTCCGAAATCGCTGATCTTTGACCCTGAGGAAATCGGGTTCGTGGTGAAAGAAACGGTCCCCATACC

AGCGAGCGGAGACTATCAGGATCTCCCCTTGTGGAGGGGACTTACGATCGCTGCGGTCAGCGAGATTCGCAG

GAATTACTCGCAGGACATCATCATCCCAATGACGCTCGTGCACCCGGACTATCTGACTGAGATACTCGACGGG

TTAAGGCAGATCGACGATCAGCTGCTGCACATCTTTCTGATGCTTAACGAGGACCTATTGCGTCACCGGATCTC

GAACCAGACCATGCATCCTGACCCGAATCGGAATGCGGAGATTCGAGAGTGGCGATTAGCGAATGTCGCCCG

ATGCTTGGCCGCAAGGGAACGGCTTCCGTCCACAACCCGTGTTCTCGATAGTGGTGCACACACCAGCGATGAA

CTTGCAGCGATGGTGCTCGACAGACTCGATCAGCGCACCTGATCGCCTTAGAGCTGGCTCGGGAAATCTGCAC

AGCGGGGATAAGTGGAGTTTCTGCCTGACTTCGGCTTAGATGCCGGGAACAGGAGAGACCATGACGAGACG

ACCGCGCCGGAACCATAGCCCGGCTTTCAAGGCGAAGGTGGCACTTGCCGCCATCCGAGGTGAGCAGACGCT

GGTGGAGTTGTCCCAGCAGTTCGATGTGCACGCCAACCAGATCAAGCAATGGAAAGACCAGCTCCTTGAGGG

GGCGACAGGTGTGTTCGGCGATGAAACGAAAGCGGAGCCGTCGGGTCCGACCATCGATGTCAAAACGCTGC

ACGCGAAAATCGGCGAACTGACACTGGAGAACGATTTTTTATCCGGTGCGCTCGGCAAGGCGGGATTGCTGG

GCGGAAAGAAATGATCGACCGCGAGCACAAGCTATCCGTCGTGCGCCAGGCGAAGCTTCTCGGCTTCAGCCG

TGGCAGCGTCTACTATCTGCCTCGTCCGGTGTCTGACGGCGATCTGGCCCTTATGCGCCGGATTGACGAATTG

CATCTCGACTACCCCTTTGCCGGAAGTCGGATGTTGCAAGGGCTCTTGAGAGGAGAAGGTCTGGAGACCGGG

CGGCGACACGTCGCCACGCTGATGAAGAAGATGGGCATCAAGGCGATCTACCGCCGCCCGAACACCTCGAAA

CCAGCGCCAGGGCACATACGAAAGCTGGCAGTCACCCGGCCCAATCAGGTGTGGGCAATGGACCTGACCTAC

ATCCCCATGGCGCGGGGATTTGTCTATCTGTGCGCCGTCGTGGACTGGTTCAGCCGGAAGGTCTTGTCATGGC

GGTTGTCGATCACGATGGAAGCAGCCTTCTGCATCGAGGCGGTGGAGGAGGCACTTGCCCGTCATGGCAGGC

CGGAAATCTTCAATACCGACCAGGGATCGCAGTTCACCTCCATCGACTTCACCGCCGTGCTGAAGAGGTCACA

GATTGCCATCTCGATGGATGGCAAGGGTGCGTGGCGAGACAATGTCTTCGTCGAGCGGCTCTGGCGTTCGAT

CAAATACGAGGAAGTCTACCTCCATGCCTACAAGACTGTGTCCGAGGCACGCGCTGGCATCGCCCGATATCTG

AACTTCTACAACACCAGACGCCCACATTGGCACTGTTGCAAAGTTAGCGATGAGGCAGCCTTTTGTCTTATTCA

AAGGCCTTACATTTCAAAAACTCTGCTTACCAGGCGCATTTCGCCCAGGGGATCACCATAATAAAATGCTGAG

GCCTGGCCTTTGCGTAGTGCACGCATCACCTCAATACCTTTGATGGTGGCGTAAGCCGTCTTCATGGATTTAAA

TCCCAGCGTGGCGCCGATTATCCGTTTCAGTTTGCCATGATCGCATTCAATCACGTTGTTCCGGTACTTAATCTG

TCGGTGTTCAACGTCAGACGGGCACCGGCCTTCGCGTTTGAGCAGAGCAAGCGCGCGACCATAGGCGGGCGC

TTTATCCGTGTTGATGAATCGCGGGATCTGCCACTTCTTCACGTTGTTGAGGATTTTACCCAGAAACCGGTATG

CAGCTTTGCTGTTACGACGGGAGGAGAGATAAAAATCGACAGTGCGGCCCCGGCTGTCGACGGCCCGGTACA

GATACGCCCAGCGGCCATTGACCTTCACGTAGGTTTCATCCATGTGCCACGGGCAAAGATCGGAAGGGTTAC

GCCAGTACCAGCGCAGCCGTTTTTCCATTTCAGGCGCATAACGCTGAACCCAGCGGTAAATCGTGGAGTGATC

GACATTCACTCCGCGTTCAGCCAGCATCTCCTGCAGCTCACGGTAACTGATGCCGTATTTGCAGTACCAGCGTA

CGGCCCACAGAATGATGTCACGCTGAAAATGCCGGCCTTTGAATGGGTTCATGTGCAGCTCCATCAGCAAAAG

GGGATGATAAGTTTATCACCACCGACTATTTGCAACAGTGCCCAAATGGTGGTCACCATCCTGTCGGCTGTGG

CACAGGCTGAACGCCGGAGGATCCTAGAACGCACGAATGAGGGCCGACAGGAAGCAAAGCTGAAAGGAATC

AAATTTGGCCGCAGGCGTACCGTGGACAGGAACGTCGTGCTGACGCTTCATCAGAAGGGCACTGGTGCAACG

GAAATTGCTCATCAGCTCAGTATTGCCCGCTCCACGGTTTATAAAATTCTTGAAGACGAAAGGGCCTCGTGAT

ACGCCTATTTTTATAGGTTAATGTCATGATAATAATGGTTTCTTAGACGTCAGGTGGCACTTTTCGGGGAAATG

TGCGCGGAACCCCTATTTGTTTATTTTTCTAAATACATTCAAATATGTATCCGCTCATGAGACAATAACCCTGGT

AAATGCTTCAATAATATTGAAAAAGGAAGAGTATGAGTATTCAACATTTCCGTGTCGCCCTTATTCCCTTTTTTG

CGGCATTTTGCCTTCCTGTTTTTGCTCACCCAGAAACGCTGGTGAAAGTAAAAGATGCTGAAGATCAGTTGGG

TGCACGAGTGGGTTACATCGAACTGGATCTCAACAGCGGTAAGATCCTTGAGAGTTTTCGCCCCGAAGAACGT

TTTCCAATGATGAGCACTTTTAAAGTTCTGCTATGTGGTGCGGTATTATCCCGTGTTGACGCCGGGCAAGAGC

AACTCGGTCGCCGCATACACTATTCTCAGAATGACTTGGTTGAGTACTCACCAGTCACAGAAAAGCATCTTACG

GATGGCATGACAGTAAGAGAATTATGCAGTGCTGCCATAACCATGAGTGATAACACTGCTGCCAACTTACTTC

TGACAACGATCGGAGGACCGAAGGAGCTAACCGCTTTTTTGCACAACATGGGGGATCATGTAACTCGCCTTG

ATCGTTGGGAACCGGAGCTGAATGAAGCCATACCAAACGACGAGCGTGACACCACGATGCCTGCAGCAATGG
CAACAACGTTGCGCAAACTATTAACTGGCGAACTACTTACTCTAGCTTCCCGGCAACAATTAATAGACTGGATG
GAGGCGGATAAAGTTGCAGGACCACTTCTGCGCTCGGCCCTTCCGGCTGGCTGGTTTATTGCTGATAAATCTG
GAGCCGGTGAGCGTGGGTCTCGCGGTATCATTGCAGCACTGGGGCCAGATGGTAAGCCCTCCCGTATCGTAG
TTATCTACACGACGGGGAGTCAGGCAACTATGGATGAACGAAATAGACAGATCGCTGAGATAGGTGCCTCAC
TGATTAAGCATTGGTAACTGTCAGACCAAGTTTACTCATATATACTTTAGATTGATTTAAAACTTCATTTTTAAT
TTAAAAGGATCTAGGTGAAGATCCTTTTTGATAATCTCATGACCAAAATCCCTTAACGTGAGTTTTCGTTCCACT
GAGCGTCAGACCCCGTTTCACACTGGTCACGCCCTCACCGACCTCAACCACCACCCCGGCACCTTCATGGCCGA
GCACCACCGGGAACAGACCTTCCGGATCATCACCGGAAAGCGTGTAGGCATCGGTATGGCATACCCCGGTGT
GACTGATTTTCACCAGCACTTCACCTTTCTTCGGCGGTGCCACATCAATTTCAACAATTTCAAGGGGTAAACCC
GGCCCGAAGGCGACTGCTGCGCGTGATTTCATAACGGTTCCTCTGTGTCTGAGGCTTATCATGGCAGGCGATA
AGCACGAATGCCGTCCACCATACCGCACAACAGCGCTGCCTGTATAGCGCCGGTCAGCAGGTGGCCGCCACC
GGTGCCGGTTTTCTGATCAGCAGACAGATTGCCAGCAGCAGACCGTACAGTGCCGTGCCAATCAGCCACACC
GTACCGTCCGCACTCTGCTGTGTCTGACTGAAAATAAAAGCAAACAGCAGCGGGCCCGCCACGCCGGTCAGA
TTGGTCAGGCTGACCAGCACACCCTGTAGTTTTCCCTGATTTGCCGCCGATGCCCCGGCAGAGATAATGCCCT
GCAATGCAGGCAGTGCAATTCCGCCGCCTGCCAGCAGGATCAGGACCGGATACACCATCCATCCGGAAGTGA
TAGCAGACATCAGTAAAAACGCGGTGGCATCGGCAATAAATCCGGCGAAAATAATGGTTTTCTCACTCAGCCG
TTTTGCCAGCGCCCCGGCAACCACCGCCTGAAACAGTGCATGCATCGCCCCGAGTCCCGCCAGTGAAAAACCG
ACCGCCGCGCTGTCCCAGGCAAAGCGGCTCTCCGTAAACAATACCCAGACAGTGGCCGGGATCTGCCCGATA
AGCTGCGCGGTAAAAAAGACAAACAGCAACAGCGCCAGAGGTTTAAGCAGTGTGATAAAGCTGATACCTGCG
CTTTCTTGTTTCTGCTCCGCCGGTTTTTCTTCTGTCTGTACCGCCGGTTTAAAGATAAAAAAGACCATCAGAAAG
GTGCAGGCATTCAGTATTGCCGCAATGACAAACGGCAGATGCGGTGAGATATCCCCCGCCAGTCCGCCGATA
GCCGGCCCGGCAATCAGCCCGGCACCAAAGGCCGCACCGAGACGGCCGAACCAGGCGGTACGCTCGCTGAC
CGCCGTGCTGTCCGCCACTACCGAAGCCGCAACCGCGCCGGTGGCACCAGTGATCCCGGAGATAATCCGCCC
GAGATACAACATCCACAGCACATTGGACAGTGCCAGCAGTGTGTAATCAAACGCGGCACCCGCCAGGGATAA
CAGCAGCACCGGTCTGCGCCCCAGCTTATCTGACCATCTGCCCAGCAGCGGAGCAAAACAGACCTGCATCACC

GCATACAGCGCCAGCAGAATGCCGTAATGGTTTGCCACATCCGCTTCCGGGAGATATTCCCGCAGCAGTGACG

GTAATACCGGCATGATCAGACCAATTCCCATCGCGTCCAGCAGTGTAATCACCAGCGCGATGACAGCGGGTTT

ATTCATTTTTCAGATCCTGTGTTAATTCCCTATCATTGATAGAGTTGGAATATATCCCTATCAATGATAGAGTGT

CAATCTGAAAATCGGAGGAGACGGTCATGGCACGGCTGAACAGAGAATCGGTTATTGATGCGGCACTGGAA

CTGCTGAATGAGACAGGGATTGACGGGCTGACGACCCGCAAGCTGGCGCAGAAGCTGGGAATAGAACAGCC

GACACTTTACTGGCATGTGAAAAATAAACGGGCGTTACTGGATGCGCTGGCGGTGGAGATCCTGGCGCGTCA

TCATGATTATTCACTGCCTGCGGCGGGGGAATCCTGGCAGTCATTTCTGCGCAATAATGCAATGAGTTTCCGC

CGGGCGCTGCTGCGTTACCGTGACGGGGCAAAAGTGCACCTCGGCACCCGCCCTGATGAAAAACAGTATGAT

ACGGTGGAAACCCAGTTACGCTTTATGACAGAAAACGGCTTTTCACTGCGCGACGGGTTATATGCGATTTCAG

CGGTCAGTCATTTTACCCTTGGTGCCGTACTGGAGCAGCAGGAGCATACTGCCGCCCTGACCGACCGCCCTGC

AGCACCGGACGAAAACCTGCCGCCGCTATTGCGGGAAGCGCTGCAGATTATGGACAGTGATGATGGTGAGC

AGGCCTTTCTGCATGGCCTGGAGAGCCTGATCCGGGGGTTTGAGGTGCAGCTTACGGCACTGTTGCAAATAG

TCGGTGGTGATAAACTTATCATCCCCTTTTGCTGATGGAGCTGCACATGAACCCATTCAAAGGCCGGCATTTTC

AGCGTGACATCATTCTGTGGGCCGTACGCTGGTACTGCAAATACGGCATCAGTTACCGTGAGCTGCAGGAGA

TGCTGGCTGAACGCGGAGTGAATGTCGATCACTCCACGATTTACCGCTGGGTTCAGCGTTATGCGCCTGAAAT

GGAAAAACGGCTGCGCTGGTACTGGCGTAACCCTTCCGATCTTTGCCCGTGGCACATGGATGAAACCTACGTG

AAGGTCAATGGCCGCTGGGCGTATCTGTACCGGGCCGTCGACAGCCGGGGCCGCACTGTCGATTTTTATCTCT

CCTCCCGTCGTAACAGCAAAGCTGCATACCGGTTTCTGGGTAAAATCCTCAACAACGTGAAGAAGTGGCAGAT

CCCGCGATTCATCAACACGGATAAAGCGCCCGCCTATGGTCGCGCGCTTGCTCTGCTCAAACGCGAAGGCCGG

TGCCCGTCTGACGTTGAACACCGACAGATTAAGTACCGGAACAACGTGATTGAATGCGATCATGGCAAACTG

AAACGGATAATCGGCGCCACGCTGGGATTTAAATCCATGAAGACGGCTTACGCCACCATCAAAGGTATTGAG

GTGATGCGTGCACTACGCAAAGGCCAGGCCTCAGCATTTTATTATGGTGATCCCCTGGGCGAAATGCGCCTGG

TAAGCAGAGTTTTTGAAATGTAAGGCCTTTGAATAAGACAAAAGGCTGCCTCATCGCTAACTTTGCAACAGTG

CCGGTACCCCATCGATGGGGGATCCTCTAGAGTCGACCCCTCGAGGGGCATGCAAGCTTGGCGTAATCATGG

TCATAGCTGTTTCCTGTGTGAAATTGTTATCCGCTCACAATTCCACACAACATACGAGCCGGAAGCATAAAGTG

TAAAGCCTGGGGTGCCTAATGAGTGAGCTAACTCACATTAATTGCGTTGCGCTCACTGCCCGCTTTCCAGTCG

GGAAACCTGTCGTGCCAGCTGCATTAATGAATCGGCCAACGCGCGGGGAGAGGCGGTTTGCGTATTGAGCTT

GGCACTGGGCCAAGCTGAATTTCTGCCATTCATCCGCTTATTATCACTTATTCAGGCGTAGCAACCAGGCGTTT

AAGGGCACCAATAACTGCCTTAAAAAATTACGCCCCGCCCTGCCACTCATCGCAGTACTGTTGTAATTCATTAA

GCATTCTGCCGACATGGAAGCCATCACAAACGGCATGATGAACCTGAATCGCCAGCGGCATCAGCACCTTGTC

GCCTTGCGTATAATATTTGCCCATGGTGAAAACGGGGGCGAAGAAGTTGTCCATATTGGCCACGTTTAAATCA

AAACTGGTGAAACTCACCCAGGGATTGGCTGAGACGAAAAACATATTCTCAATAAACCCTTTAGGGAAATAG

GCCAGGTTTTCACCGTAACACGCCACATCTTGCGAATATATGTGTAGAAACTGCCGGAAATCGTCGTGGTATT

CACTCCAGAGCGATGAAAACGTTTCAGTTTGCTCATGGAAAACGGTGTAACAAGGGTGAACACTATCCCATAT

CACCAGCTCACCGTCTTTCATTGCCATACGAAATTCCGGATGAGCATTCATCAGGCGGGCAAGAATGTGAATA

AAGGCCGGATAAAACTTGTGCTTATTTTTCTTTACGGTCTTTAAAAAGGCCGTAATATCCAGCTGAACGGTCTG

GTTATAGGTACATTGAGCAACTGACTGAAATGCCTCAAAATGTTCTTTACGATGCCATTGGGATATATCAACG

GTGGTATATCCAGTGATTTTTTTCTCCATTTTAGCTTCCTTAGCTCCTGAAAATCTCGATAACTCAAAAAATACG

CCCGGTAGTGATCTTATTTCATTATGGTGAAAGTTGGAACCTCTTACGTGCCGATCAACGTCTCATTTTCGCCA

AAAGTTGGCCCAGGGCTTCCCGGTATCAACAGGGACACCAGGATTTATTTATTCTGCGAAGTGATCTTCCGTTC

GACGGAGTTCCACTGAGCGTCAGACCCCGTAGAAAAGATCAAAGGATCTTCTTGAGATCCTTTTTTTCTGCGCG

TAATCTGCTGCTTGCAAACAAAAAAACCACCGCTACCAGCGGTGGTTTGTTTGCCGGATCAAGAGCTACCAAC

TCTTTTTCCGAAGGTAACTGGCTTCAGCAGAGCGCAGATACCAAATACTGTTCTTCTAGTGTAGCCGTAGTTAG

GCCACCACTTCAAGAACTCTGTAGCACCGCCTACATACCTCGCTCTGCTAATCCTGTTACCAGTGGCTGCTGCC

AGTGGCGATAAGTCGTGTCTTACCGGGTTGGACTCAAGACGATAGTTACCGGATAAGGCGCAGCGGTCGGGC

TGAACGGGGGGTTCGTGCACACAGCCCAGCTTGGAGCGAACGACCTACACCGAACTGAGATACCTACAGCGT

GAGCTTATGAGAAAGCGCCACGCTTCCCGAAGGGAGAAAGGCGGACAGGTATCCGGTAAGCGGCAGGGTCG

GAACAGGAGAGCGCACGAGGGAGCTTCCAGGGGGAAACGCCTGGTATCTTTATAGTCCTGTCGGGTTTCGCC

ACCTCTGACTTGAGCGTCGATTTTTGTGATGCTCGTCAGGGGGGCGGAGCCTATGGAAAAACGCCAGCAACG

CGGCCTTTTTACGGTTCCTGGCCTTTTGCTGGCCTTTTGCTCACATGTTCTTTCCTGCGTTATCCCCTGATTCTGT

GGATAACCGTATTACCGCCTTTGAGTGAGCTGATACCGCTCGCCGCAGCCGAACGACCGAGCGCAGCGAGTC

AGTGAGCGAGGAAGCGGAAGAAGCTCATTCGCCATTCAGGCTGCGCAACTGTTGGGAAGGGCGATCGGTGC

GGGCCTCTTCGCTATTACGCCAGCTGGCGAAAGGGGGATGTGCTGCAAGGCGATTAAGTTGGGTAACGCCAG

GGTTTTCCCAGTCACGACGTTGTAAAACGACGGCCAGTGAATTC

C

GGCACTGTTGCAAATAGTCGGTGGTGATAAACTTATCATCCCCTTTTGCTGATGGAGCTGCACATGAACCCATT

CAAAGGCCGGCATTTTCAGCGTGACATCATTCTGTGGGCCGTACGCTGGTACTGCAAATACGGCATCAGTTAC

CGTGAGCTGCAGGAGATGCTGGCTGAACGCGGAGTGAATGTCGATCACTCCACGATTTACCGCTGGGTTCAG

CGTTATGCGCCTGAAATGGAAAAACGGCTGCGCTGGTACTGGCGTAACCCTTCCGATCTTTGCCCGTGGCACA

TGGATGAAACCTACGTGAAGGTCAATGGCCGCTGGGCGTATCTGTACCGGGCCGTCGACAGCCGGGGCCGCA

CTGTCGATTTTTATCTCTCCTCCCGTCGTAACAGCAAAGCTGCATACCGGTTTCTGGGTAAAATCCTCAACAAC

GTGAAGAAGTGGCAGATCCCGCGATTCATCAACACGGATAAAGCGCCCGCCTATGGTCGCGCGCTTGCTCTG

CTCAAACGCGAAGGCCGGTGCCCGTCTGACGTTGAACACCGACAGATTAAGTACCGGAACAACGTGATTGAA

TGCGATCATGGCAAACTGAAACGGATAATCGGCGCCACGCTGGGATTTAAATCCATGAAGACGGCTTACGCC

ACCATCAAAGGTATTGAGGTGATGCGTGCACTACGCAAAGGCCAGGCCTCAGCATTTTATTATGGTGATCCCC

TGGGCGAAATGCGCCTGGTAAGCAGAGTTTTTGAAATGTAAGGCCTTTGAATAAGACAAAAGGCTGCCTCAT

CGCTAACTTTGCAACAGTGCCTTTAAGCGTGCATAATAAGCCCTACACAAATTGGGAGTTAGACATCATGAGC

AACGCAAAAACAAAGTTAGGCATCACAAAGTACAGCATCGTGACCAACAGCAACGATTCCGTCACACTGCGCC

TCATGACTGAGCATGACCTTGCGATGCTCTATGAGTGGCTAAATCGATCTCATATCGTCGAGTGGTGGGGCGG

AGAAGAAGCACGCCCGACACTTGCTGACGTACAGGAACAGTACTTGCCAAGCGTTTTAGCGCAAGAGTCCGT

CACTCCATACATTGCAATGCTGAATGGAGAGCCGATTGGGTATGCCCAGTCGTACGTTGCTCTTGGAAGCGGG

GACGGACGGTGGGAAGAAGAAACCGATCCAGGAGTACGCGGAATAGACCAGTTACTGGCGAATGCATCACA

ACTGGGCAAAGGCTTGGGAACCAAGCTGGTTCGAGCTCTGGTTGAGTTGCTGTTCAATGATCCCGAGGTCACC

AAGATCCAAACGGACCCGTCGCCGAGCAACTTGCGAGCGATCCGATGCTACGAGAAAGCGGGGTTTGAGAG

GCAAGGTACCGTAACCACCCCATATGGTCCAGCCGTGTACATGGTTCAAACACGCCAGGCATTCGAGCGAACA

CGCAGTGATGCCTAACCCTTCCATCGAGGGGGACGTCCAAGGGCTGGCGCCCTTGGCCGCCCCTCATGTCAAA

CGTTGGGCGAACCCGGAGCCTCATTAATTGTTAGCCGTTAAAATTAAGCCCTTTACCAAACCAATACTTATTAT

GAAAAACACAATACATATCAACTTCGCTATTTTTTTAATAATTGCAAATATTATCTACAGCAGCGCCAGTGCATC

AACAGATATCTCTACTGTTGCATCTCCATTATTTGAAGGAACTGAAGGTTGTTTTTTACTTTACGATGCATCCAC

AAACGCTGAAATTGCTCAATTCAATAAAGCAAAGTGTGCAACGCAAATGGCACCAGATTCAACTTTCAAGATC

GCATTATCACTTATGGCATTTGATGCGGAAATAATAGATCAGAAAACCATATTCAAATGGGATAAAACCCCCA

AAGGAATGGAGATCTGGAACAGCAATCATACACCAAAGACGTGGATGCAATTTTCTGTTGTTTGGGTTTCGCA

AGAAATAACCCAAAAAATTGGATTAAATAAAATCAAGAATTATCTCAAAGATTTTGATTATGGAAATCAAGAC

TTCTCTGGAGATAAAGAAAGAAACAACGGATTAACAGAAGCATGGCTCGAAAGTAGCTTAAAAAATTTCACC

AGAAGAACAAATTCAATTCCTGCGTAAAATTATTAATCACAATCTCCCAGTTAAAAACTCAGCCATAGAAAACA

CCATAGAGAACATGTATCTACAAGATCTGGATAATAGTACAAAACTGTATGGGAAAACTGGTGCAGGATTCAC

AGCAAATAGAACCTTACAAAACGGATGGTTTGAAGGGTTTATTATAAGCAAATCAGGACATAAATATGTTTTT

GTGTCCGCACTTACAGGAAACTTGGGGTCGAATTTAACATCAAGCATAAAAGCCAAGAAAAATGCGATCACCA

TTCTAAACACACTAAATTTATAAAAAATCTAATGGCAAAATCGCCCAACCCTTCAATCAAGTCGGGACGGCCAA

AAGCAAGCTTTTGGCTCCCCTCGCTGGCGCTCGGCGCCCCTTATTTCAAACGTTAGACGGCAAAGTCACAGAC

CGCGGGATCTCTTATGACCAACTACTTTGATAGCCCCTTCAAAGGCAAGCTGCTTTCTGAGCAAGTGAAGAAC

CCCAATATCAAAGTTGGGCGGTACAGCTATTACTCTGGCTACTATCATGGGCACTCATTCGATGACTGCGCAC

GGTATCTGTTTCCGGACCGTGATGACGTTGATAAGTTGATCATCGGTAGTTTCTGCTCTATCGGGAGTGGGGC

TTCCTTTATCATGGCTGGCAATCAGGGGCATCGGTACGACTGGGCATCATCTTTCCCGTTCTTTTATATGCAGG

AAGAACCTGCATTCTCAAGCGCACTCGATGCCTTCCAAAAAGCAGGTAATACTGTCATTGGCAATGACGTTTG

GATCGGCTCTGAGGCAATGGTCATGCCCGGAATCAAGATCGGGCACGGTGCGGTGATAGGCAGCCGCTCGTT

GGTGACAAAAGATGTGGGGCACTGTTGCAAAGTTAGCGATGAGGCAGCCTTTTGTCTTATTCAAAGGCCTTAC

ATTTCAAAAACTCTGCTTACCAGGCGCATTTCGCCCAGGGGATCACCATAATAAAATGCTGAGGCCTGGCCTTT

GCGTAGTGCACGCATCACCTCAATACCTTTGATGGTGGCGTAAGCCGTCTTCATGGATTTAAATCCCAGCGTG

GCGCCGATTATCCGTTTCAGTTTGCCATGATCGCATTCAATCACGTTGTTCCGGTACTTAATCTGTCGGTGTTCA

ACGTCAGACGGGCACCGGCCTTCGCGTTTGAGCAGAGCAAGCGCGCGACCATAGGCGGGCGCTTTATCCGTG

TTGATGAATCGCGGGATCTGCCACTTCTTCACGTTGTTGAGGATTTTACCCAGAAACCGGTATGCAGCTTTGCT

GTTACGACGGGAGGAGAGATAAAAATCGACAGTGCGGCCCCGGCTGTCGACGGCCCGGTACAGATACGCCC

AGCGGCCATTGACCTTCACGTAGGTTTCATCCATGTGCCACGGGCAAAGATCGGAAGGGTTACGCCAGTACCA

GCGCAGCCGTTTTTCCATTTCAGGCGCATAACGCTGAACCCAGCGGTAAATCGTGGAGTGATCGACATTCACT

CCGCGTTCAGCCAGCATCTCCTGCAGCTCACGGTAACTGATGCCGTATTTGCAGTACCAGCGTACGGCCCACA

GAATGATGTCACGCTGAAAATGCCGGCCTTTGAATGGGTTCATGTGCAGCTCCATCAGCAAAAGGGGATGAT

AAGTTTATCACCACCGACTATTTGCAACAGTGCCTCTCGATAGGTATAGTGTTTTGCAGTTTAGAGGAGATATC

GCGATGCATACGCGGAAGGCAATAACGGAGGCAATTCGAAAATTAGGAGTCCAAACCGGTGACCTGTTGATG

GTGCATGCCTCACTTAAAGCGATTGGTCCGGTCGAAGGAGGAGCGGAGACGGTCGTTGCCGCGTTACGCTCC

GCGGTTGGGCCGACTGGCACTGTGATGGGATACGCGTCGTGGGACCGATCACCCTACGAGGAGACTCTGAAT

GGCGCTCGGTTGGATGACAAAGCCCGCCGTACCTGGCCGCCGTTCGATCCCGCAACGGCCGGGACTTACCGT

GGGTTCGGCCTGCTGAATCAATTTCTGGTTCAAGCCCCCGGCGCGCGGCGCAGCGCGCACCCCGATGCATCG

ATGGTCGCGGTTGGTCCGCTAGCTGAAACGCTGACGGAGCCTCACGAACTCGGTCACGCCTTGGGGGAAGGG

TCGCCCGTCGAGCGGTTCGTCCGCCTTGGCGGGAAGGCCCTGCTGTTGGGTGCGCCGCTAAACTCCGTTACCG

CATTGCACTACGCCGAGGCGGTTGCGGATATCCCCAACAAACGATGGGTGACGTATGAGATGCCGATGCTTG

GAAGAAACGGTGAAGTCGCCTGGAAAACGGCATCAGAATACGATTCAAACGGCATTCTCGATTGCTTTGCTAT

CGAAGGAAAGCCGGATGCGGTCGAAACTATAGCAAATGCTTACGTGAAGCTCGGTCGCCATCGAGAAGGTGT

CGTGGGCTTTGCTCAGTGCTACCTGTTCGACGCGCAGGACATCGTGACGTTCGGCGTCACCTATCTTGAGAAG

CACTTCGGAGCCACTCCGATCGTGCCAGCACACGAAGCCGCCCAGCGCTCTTGCGAGCCTTCCGGTTAGAGGC

CGTCGACAATGATAATCTGGATCAACGGACCTTTCGGCGCCGGAAAGACGACTCTCGCTGAGCGGCTGCGCG

ATCGGCGTCCGAAATCGCTGATCTTTGACCCTGAGGAAATCGGGTTCGTGGTGAAAGAAACGGTCCCCATACC

AGCGAGCGGAGACTATCAGGATCTCCCCTTGTGGAGGGGACTTACGATCGCTGCGGTCAGCGAGATTCGCAG

GAATTACTCGCAGGACATCATCATCCCAATGACGCTCGTGCACCCGGACTATCTGACTGAGATACTCGACGGG

TTAAGGCAGATCGACGATCAGCTGCTGCACATCTTTCTGATGCTTAACGAGGACCTATTGCGTCACCGGATCTC

GAACCAGACCATGCATCCTGACCCGAATCGGAATGCGGAGATTCGAGAGTGGCGATTAGCGAATGTCGCCCG

ATGCTTGGCCGCAAGGGAACGGCTTCCGTCCACAACCCGTGTTCTCGATAGTGGTGCACACACCAGCGATGAA

CTTGCAGCGATGGTGCTCGACAGACTCGATCAGCGCACCTGATCGCCTTAGAGCTGGCTCGGGAAATCTGCAC

AGCGGGGATAAGTGGAGTTTCTGCCTGACTTCGGCTTAGATGCCGGGAACAGGAGAGACCATGACGAGACG

ACCGCGCCGGAACCATAGCCCGGCTTTCAAGGCGAAGGTGGCACTTGCCGCCATCCGAGGTGAGCAGACGCT

GGTGGAGTTGTCCCAGCAGTTCGATGTGCACGCCAACCAGATCAAGCAATGGAAAGACCAGCTCCTTGAGGG

GGCGACAGGTGTGTTCGGCGATGAAACGAAAGCGGAGCCGTCGGGTCCGACCATCGATGTCAAAACGCTGC

ACGCGAAAATCGGCGAACTGACACTGGAGAACGATTTTTTATCCGGTGCGCTCGGCAAGGCGGGATTGCTGG

GCGGAAAGAAATGATCGACCGCGAGCACAAGCTATCCGTCGTGCGCCAGGCGAAGCTTCTCGGCTTCAGCCG

TGGCAGCGTCTACTATCTGCCTCGTCCGGTGTCTGACGGCGATCTGGCCCTTATGCGCCGGATTGACGAATTG

CATCTCGACTACCCCTTTGCCGGAAGTCGGATGTTGCAAGGGCTCTTGAGAGGAGAAGGTCTGGAGACCGGG

CGGCGACACGTCGCCACGCTGATGAAGAAGATGGGCATCAAGGCGATCTACCGCCGCCCGAACACCTCGAAA

CCAGCGCCAGGGCACATACGAAAGCTGGCAGTCACCCGGCCCAATCAGGTGTGGGCAATGGACCTGACCTAC

ATCCCCATGGCGCGGGGATTTGTCTATCTGTGCGCCGTCGTGGACTGGTTCAGCCGGAAGGTCTTGTCATGGC

GGTTGTCGATCACGATGGAAGCAGCCTTCTGCATCGAGGCGGTGGAGGAGGCACTTGCCCGTCATGGCAGGC

CGGAAATCTTCAATACCGACCAGGGATCGCAGTTCACCTCCATCGACTTCACCGCCGTGCTGAAGAGGTCACA

GATTGCCATCTCGATGGATGGCAAGGGTGCGTGGCGAGACAATGTCTTCGTCGAGCGGCTCTGGCGTTCGAT

CAAATACGAGGAAGTCTACCTCCATGCCTACAAGACTGTGTCCGAGGCACGCGCTGGCATCGCCCGATATCTG

AACTTCTACAACACCAGACGCCCACATTGGCACTGTTGCAAAGTTAGCGATGAGGCAGCCTTTTGTCTTATTCA

AAGGCCTTACATTTCAAAAACTCTGCTTACCAGGCGCATTTCGCCCAGGGGATCACCATAATAAAATGCTGAG

GCCTGGCCTTTGCGTAGTGCACGCATCACCTCAATACCTTTGATGGTGGCGTAAGCCGTCTTCATGGATTTAAA

TCCCAGCGTGGCGCCGATTATCCGTTTCAGTTTGCCATGATCGCATTCAATCACGTTGTTCCGGTACTTAATCTG

TCGGTGTTCAACGTCAGACGGGCACCGGCCTTCGCGTTTGAGCAGAGCAAGCGCGCGACCATAGGCGGGCGC

TTTATCCGTGTTGATGAATCGCGGGATCTGCCACTTCTTCACGTTGTTGAGGATTTTACCCAGAAACCGGTATG

CAGCTTTGCTGTTACGACGGGAGGAGAGATAAAAATCGACAGTGCGGCCCCGGCTGTCGACGGCCCGGTACA

GATACGCCCAGCGGCCATTGACCTTCACGTAGGTTTCATCCATGTGCCACGGGCAAAGATCGGAAGGGTTAC

GCCAGTACCAGCGCAGCCGTTTTTCCATTTCAGGCGCATAACGCTGAACCCAGCGGTAAATCGTGGAGTGATC

GACATTCACTCCGCGTTCAGCCAGCATCTCCTGCAGCTCACGGTAACTGATGCCGTATTTGCAGTACCAGCGTA

CGGCCCACAGAATGATGTCACGCTGAAAATGCCGGCCTTTGAATGGGTTCATGTGCAGCTCCATCAGCAAAAG

GGGATGATAAGTTTATCACCACCGACTATTTGCAACAGTGCCCAAATGGTGGTCACCATCCTGTCGGCTGTGG

CACAGGCTGAACGCCGGAGGATCCTAGAACGCACGAATGAGGGCCGACAGGAAGCAAAGCTGAAAGGAATC

AAATTTGGCCGCAGGCGTACCGTGGACAGGAACGTCGTGCTGACGCTTCATCAGAAGGGCACTGGTGCAACG

GAAATTGCTCATCAGCTCAGTATTGCCCGCTCCACGGTTTATAAAATTCTTGAAGACGAAAGGGCCTCGTGAT

ACGCCTATTTTTATAGGTTAATGTCATGATAATAATGGTTTCTTAGACGTCAGGTGGCACTTTTCGGGGAAATG

TGCGCGGAACCCCTATTTGTTTATTTTTCTAAATACATTCAAATATGTATCCGCTCATGAGACAATAACCCTGGT

AAATGCTTCAATAATATTGAAAAAGGAAGAGTATGAGTATTCAACATTTCGTGTCGCCCTTATTCCCTTTTTTG

CGGCATTTTGCCTTCCTGTTTTTGCTCACCCAGAAACGCTGGTGAAAGTAAAAGATGCTGAAGATCAGTTGGG

TGCACGAGTGGGTTACATCGAACTGGATCTCAACAGCGGTAAGATCCTTGAGAGTTTTCGCCCCGAAGAACGT

TTTCCAATGATGAGCACTTTTAAAGTTCTGCTATGTGGTGCGGTATTATCCCGTGTTGACGCCGGGCAAGAGC

AACTCGGTCGCCGCATACACTATTCTCAGAATGACTTGGTTGAGTACTCACCAGTCACAGAAAAGCATCTTACG

GATGGCATGACAGTAAGAGAATTATGCAGTGCTGCCATAACCATGAGTGATAACACTGCTGCCAACTTACTTC

TGACAACGATCGGAGGACCGAAGGAGCTAACCGCTTTTTTGCACAACATGGGGGATCATGTAACTCGCCTTG

ATCGTTGGGAACCGGAGCTGAATGAAGCCATACCAAACGACGAGCGTGACACCACGATGCCTGCAGCAATGG

CAACAACGTTGCGCAAACTATTAACTGGCGAACTACTTACTCTAGCTTCCCGGCAACAATTAATAGACTGGATG

GAGGCGGATAAAGTTGCAGGACCACTTCTGCGCTCGGCCCTTCCGGCTGGCTGGTTTATTGCTGATAAATCTG

GAGCCGGTGAGCGTGGGTCTCGCGGTATCATTGCAGCACTGGGGCCAGATGGTAAGCCCTCCCGTATCGTAG

TTATCTACACGACGGGGAGTCAGGCAACTATGGATGAACGAAATAGACAGATCGCTGAGATAGGTGCCTCAC

TGATTAAGCATTGGTAACTGTCAGACCAAGTTTACTCATATATACTTTAGATTGATTTAAAACTTCATTTTTAAT

TTAAAAGGATCTAGGTGAAGATCCTTTTTGATAATCTCATGACCAAAATCCCTTAACGTGAGTTTTCGTTCCACT

GAGCGTCAGACCCCGTTTCACACTGGTCACGCCCTCACCGACCTCAACCACCACCCCGGCACCTTCATGGCCGA

GCACCACCGGGAACAGACCTTCCGGATCATCACCGGAAAGCGTGTAGGCATCGGTATGGCATACCCCGGTGT

GACTGATTTTCACCAGCACTTCACCTTTCTTCGGCGGTGCCACATCAATTTCAACAATTTCAAGGGGTAAACCC

GGCCCGAAGGCGACTGCTGCGCGTGATTTCATAACGGTTCCTCTGTGTCTGAGGCTTATCATGGCAGGCGATA

AGCACGAATGCCGTCCACCATACCGCACAACAGCGCTGCCTGTATAGCGCCGGTCAGCAGGTGGCCGCCACC

GGTGCCGGTTTTCTGATCAGCAGACAGATTGCCAGCAGCAGACCGTACAGTGCCGTGCCAATCAGCCACACC

GTACCGTCCGCACTCTGCTGTGTCTGACTGAAAATAAAAGCAAACAGCAGCGGGCCCGCCACGCCGGTCAGA

TTGGTCAGGCTGACCAGCACACCCTGTAGTTTTCCCTGATTTGCCGCCGATGCCCCGGCAGAGATAATGCCCT

GCAATGCAGGCAGTGCAATTCCGCCGCCTGCCAGCAGGATCAGGACCGGATACACCATCCATCCGGAAGTGA

TAGCAGACATCAGTAAAAACGCGGTGGCATCGGCAATAAATCCGGCGAAAATAATGGTTTTCTCACTCAGCCG

TTTTGCCAGCGCCCCGGCAACCACCGCCTGAAACAGTGCATGCATCGCCCCGAGTCCCGCCAGTGAAAAACCG

ACCGCCGCGCTGTCCCAGGCAAAGCGGCTCTCCGTAAACAATACCCAGACAGTGGCCGGGATCTGCCCGATA

AGCTGCGCGGTAAAAAAGACAAACAGCAACAGCGCCAGAGGTTTAAGCAGTGTGATAAAGCTGATACCTGCG

CTTTCTTGTTTCTGCTCCGCCGGTTTTTCTTCTGTCTGTACCGCCGGTTTAAAGATAAAAAAGACCATCAGAAAG

GTGCAGGCATTCAGTATTGCCGCAATGACAAACGGCAGATGCGGTGAGATATCCCCCGCCAGTCCGCCGATA

GCCGGCCCGGCAATCAGCCCGGCACCAAAGGCCGCACCGAGACGGCCGAACCAGGCGGTACGCTCGCTGAC

CGCCGTGCTGTCCGCCACTACCGAAGCCGCAACCGCGCCGGTGGCACCAGTGATCCCGGAGATAATCCGCCC

GAGATACAACATCCACAGCACATTGGACAGTGCCAGCAGTGTGTAATCAAACGCGGCACCCGCCAGGGATAA

CAGCAGCACCGGTCTGCGCCCCAGCTTATCTGACCATCTGCCCAGCAGCGGAGCAAAACAGACCTGCATCACC

GCATACAGCGCCAGCAGAATGCCGTAATGGTTTGCCACATCCGCTTCCGGGAGATATTCCCGCAGCAGTGACG

GTAATACCGGCATGATCAGACCAATTCCCATCGCGTCCAGCAGTGTAATCACCAGCGCGATGACAGCGGGTTT

ATTCATTTTTCAGATCCTGTGTTAATTCCCTATCATTGATAGAGTTGGAATATATCCCTATCAATGATAGAGTGT

CAATCTGAAAATCGGAGGAGACGGTCATGGCACGGCTGAACAGAGAATCGGTTATTGATGCGGCACTGGAA

CTGCTGAATGAGACAGGGATTGACGGGCTGACGACCCGCAAGCTGGCGCAGAAGCTGGGAATAGAACAGCC

GACACTTTACTGGCATGTGAAAAATAAACGGGCGTTACTGGATGCGCTGGCGGTGGAGATCCTGGCGCGTCA

TCATGATTATTCACTGCCTGCGGCGGGGGAATCCTGGCAGTCATTTCTGCGCAATAATGCAATGAGTTTCCGC

CGGGCGCTGCTGCGTTACCGTGACGGGGCAAAAGTGCACCTCGGCACCCGCCCTGATGAAAAACAGTATGAT

ACGGTGGAAACCCAGTTACGCTTTATGACAGAAAACGGCTTTTCACTGCGCGACGGGTTATATGCGATTTCAG

CGGTCAGTCATTTTACCCTTGGTGCCGTACTGGAGCAGCAGGAGCATACTGCCGCCCTGACCGACCGCCCTGC

AGCACCGGACGAAAACCTGCCGCCGCTATTGCGGGAAGCGCTGCAGATTATGGACAGTGATGATGGTGAGC

AGGCCTTTCTGCATGGCCTGGAGAGCCTGATCCGGGGGTTTGAGGTGCAGCTTACGGCACTGTTGCAAATAG

TCGGTGGTGATAAACTTATCATCCCCTTTTGCTGATGGAGCTGCACATGAACCCATTCAAAGGCCGGCATTTTC

AGCGTGACATCATTCTGTGGGCCGTACGCTGGTACTGCAAATACGGCATCAGTTACCGTGAGCTGCAGGAGA

TGCTGGCTGAACGCGGAGTGAATGTCGATCACTCCACGATTTACCGCTGGGTTCAGCGTTATGCGCCTGAAAT

GGAAAAACGGCTGCGCTGGTACTGGCGTAACCCTTCCGATCTTTGCCCGTGGCACATGGATGAAACCTACGTG

AAGGTCAATGGCCGCTGGGCGTATCTGTACCGGGCCGTCGACAGCCGGGGCCGCACTGTCGATTTTTATCTCT

CCTCCCGTCGTAACAGCAAAGCTGCATACCGGTTTCTGGGTAAAATCCTCAACAACGTGAAGAAGTGGCAGAT

CCCGCGATTCATCAACACGGATAAAGCGCCCGCCTATGGTCGCGCGCTTGCTCTGCTCAAACGCGAAGGCCGG

TGCCCGTCTGACGTTGAACACCGACAGATTAAGTACCGGAACAACGTGATTGAATGCGATCATGGCAAACTG

AAACGGATAATCGGCGCCACGCTGGGATTTAAATCCATGAAGACGGCTTACGCCACCATCAAAGGTATTGAG

GTGATGCGTGCACTACGCAAAGGCCAGGCCTCAGCATTTTATTATGGTGATCCCCTGGGCGAAATGCGCCTGG

TAAGCAGAGTTTTTGAAATGTAAGGCCTTTGAATAAGACAAAAGGCTGCCTCATCGCTAACTTTGCAACAGTG

CCGGTACCCCATCGATGGGGGATCCTCTAGAGTCGACCCCTCGAGGGGCATGCAAGCTTGGCGTAATCATGG

TCATAGCTGTTTCCTGTGTGAAATTGTTATCCGCTCACAATTCCACACAACATACGAGCCGGAAGCATAAAGTG

TAAAGCCTGGGGTGCCTAATGAGTGAGCTAACTCACATTAATTGCGTTGCGCTCACTGCCCGCTTTCCAGTCG

GGAAACCTGTCGTGCCAGCTGCATTAATGAATCGGCCAACGCGCGGGGAGAGGCGGTTTGCGTATTGAGCTT

GGCACTGGGCCAAGCTGAATTTCTGCCATTCATCCGCTTATTATCACTTATTCAGGCGTAGCAACCAGGCGTTT

AAGGGCACCAATAACTGCCTTAAAAAATTACGCCCCGCCCTGCCACTCATCGCAGTACTGTTGTAATTCATTAA

GCATTCTGCCGACATGGAAGCCATCACAAACGGCATGATGAACCTGAATCGCCAGCGGCATCAGCACCTTGTC

GCCTTGCGTATAATATTTGCCCATGGTGAAAACGGGGGCGAAGAAGTTGTCCATATTGGCCACGTTTAAATCA

AAACTGGTGAAACTCACCCAGGGATTGGCTGAGACGAAAAACATATTCTCAATAAACCCTTTAGGGAAATAG

GCCAGGTTTTCACCGTAACACGCCACATCTTGCGAATATATGTGTAGAAACTGCCGGAAATCGTCGTGGTATT

CACTCCAGAGCGATGAAAACGTTTCAGTTTGCTCATGGAAAACGGTGTAACAAGGGTGAACACTATCCCATAT

CACCAGCTCACCGTCTTTCATTGCCATACGAAATTCCGGATGAGCATTCATCAGGCGGGCAAGAATGTGAATA

AAGGCCGGATAAAACTTGTGCTTATTTTTCTTTACGGTCTTTAAAAAGGCCGTAATATCCAGCTGAACGGTCTG

GTTATAGGTACATTGAGCAACTGACTGAAATGCCTCAAAATGTTCTTTACGATGCCATTGGGATATATCAACG

GTGGTATATCCAGTGATTTTTTTCTCCATTTTAGCTTCCTTAGCTCCTGAAAATCTCGATAACTCAAAAAATACG

CCCGGTAGTGATCTTATTTCATTATGGTGAAAGTTGGAACCTCTTACGTGCCGATCAACGTCTCATTTTCGCCA

AAAGTTGGCCCAGGGCTTCCCGGTATCAACAGGGACACCAGGATTTATTTATTCTGCGAAGTGATCTTCCGTTC

GACGGAGTTCCACTGAGCGTCAGACCCCGTAGAAAAGATCAAAGGATCTTCTTGAGATCCTTTTTTCTGCGCG

TAATCTGCTGCTTGCAAACAAAAAAACCACCGCTACCAGCGGTGGTTTGTTTGCCGGATCAAGAGCTACCAAC

TCTTTTTCCGAAGGTAACTGGCTTCAGCAGAGCGCAGATACCAAATACTGTTCTTCTAGTGTAGCCGTAGTTAG

GCCACCACTTCAAGAACTCTGTAGCACCGCCTACATACCTCGCTCTGCTAATCCTGTTACCAGTGGCTGCTGCC

AGTGGCGATAAGTCGTGTCTTACCGGGTTGGACTCAAGACGATAGTTACCGGATAAGGCGCAGCGGTCGGGC

TGAACGGGGGGTTCGTGCACACAGCCCAGCTTGGAGCGAACGACCTACACCGAACTGAGATACCTACAGCGT

GAGCTATGAGAAAGCGCCACGCTTCCCGAAGGGAGAAAGGCGGACAGGTATCCGGTAAGCGGCAGGGTCG

GAACAGGAGAGCGCACGAGGGAGCTTCCAGGGGGAAACGCCTGGTATCTTTATAGTCCTGTCGGGTTTCGCC

ACCTCTGACTTGAGCGTCGATTTTTGTGATGCTCGTCAGGGGGGCGGAGCCTATGGAAAAACGCCAGCAACGC

GGCCTTTTTACGGTTCCTGGCCTTTTGCTGGCCTTTTGCTCACATGTTCTTTCCTGCGTTATCCCCTGATTCTGTG

GATAACCGTATTACCGCCTTTGAGTGAGCTGATACCGCTCGCCGCAGCCGAACGACCGAGCGCAGCGAGTCA

GTGAGCGAGGAAGCGGAAGAAGCTCATTCGCCATTCAGGCTGCGCAACTGTTGGGAAGGGCGATCGGTGCG

GGCCTCTTCGCTATTACGCCAGCTGGCGAAAGGGGGATGTGCTGCAAGGCGATTAAGTTGGGTAACGCCAGG

GTTTTCCCAGTCACGACGTTGTAAAACGACGGCCAGTGAATTCGGCACTGTTGCAAATAGTCGGTGGTGATAA

ACTTATCATCCCCTTTTGCTGATGGAGCTGCACATGAACCCATTCAAAGGCCGGCATTTTCAGCGTGACATCAT

TCTGTGGGCCGTACGCTGGTACTGCAAATACGGCATCAGTTACCGTGAGCTGCAGGAGATGCTGGCTGAACG

CGGAGTGAATGTCGATCACTCCACGATTTACCGCTGGGTTCAGCGTTATGCGCCTGAAATGGAAAAACGGCTG

CGCTGGTACTGGCGTAACCCTTCCGATCTTTGCCCGTGGCACATGGATGAAACCTACGTGAAGGTCAATGGCC

GCTGGGCGTATCTGTACCGGGCCGTCGACAGCCGGGGCCGCACTGTCGATTTTTATCTCTCCTCCCGTCGTAA

CAGCAAAGCTGCATACCGGTTTCTGGGTAAAATCCTCAACAACGTGAAGAAGTGGCAGATCCCGCGATTCATC

AACACGGATAAAGCGCCCGCCTATGGTCGCGCGCTTGCTCTGCTCAAACGCGAAGGCCGGTGCCCGTCTGAC

GTTGAACACCGACAGATTAAGTACCGGAACAACGTGATTGAATGCGATCATGGCAAACTGAAACGGATAATC

GGCGCCACGCTGGGATTTAAATCCATGAAGACGGCTTACGCCACCATCAAAGGTATTGAGGTGATGCGTGCA

CTACGCAAAGGCCAGGCCTCAGCATTTTATTATGGTGATCCCCTGGGCGAAATGCGCCTGGTAAGCAGAGTTT

TTGAAATGTAAGGCCTTTGAATAAGACAAAAGGCTGCCTCATCGCTAACTTTGCAACAGTGCCTTTAAGCGTG

CATAATAAGCCCTACACAAATTGGGAGTTAGACATCATGAGCAACGCAAAAACAAAGTTAGGCATCACAAAG

TACAGCATCGTGACCAACAGCAACGATTCCGTCACACTGCGCCTCATGACTGAGCATGACCTTGCGATGCTCT

ATGAGTGGCTAAATCGATCTCATATCGTCGAGTGGTGGGGCGGAGAAGAAGCACGCCCGACACTTGCTGACG

TACAGGAACAGTACTTGCCAAGCGTTTTAGCGCAAGAGTCCGTCACTCCATACATTGCAATGCTGAATGGAGA

GCCGATTGGGTATGCCCAGTCGTACGTTGCTCTTGGAAGCGGGGACGGACGGTGGGAAGAAGAAACCGATC

CAGGAGTACGCGGAATAGACCAGTTACTGGCGAATGCATCACAACTGGGCAAAGGCTTGGGAACCAAGCTG

GTTCGAGCTCTGGTTGAGTTGCTGTTCAATGATCCCGAGGTCACCAAGATCCAAACGGACCCGTCGCCGAGCA

ACTTGCGAGCGATCCGATGCTACGAGAAAGCGGGGTTTGAGAGGCAAGGTACCGTAACCACCCCATATGGTC

CAGCCGTGTACATGGTTCAAACACGCCAGGCATTCGAGCGAACACGCAGTGATGCCTAACCCTTCCATCGAGG

GGGACGTCCAAGGGCTGGCGCCCTTGGCCGCCCCTCATGTCAAACGTTGGGCGAACCCGGAGCCTCATTAATT

GTTAGCCGTTAAAATTAAGCCCTTTACCAAACCAATACTTATTATGAAAAACACAATACATATCAACTTCGCTAT

TTTTTTAATAATTGCAAATATTATCTACAGCAGCGCCAGTGCATCAACAGATATCTCTACTGTTGCATCTCCATT

ATTTGAAGGAACTGAAGGTTGTTTTTACTTTACGATGCATCCACAAACGCTGAAATTGCTCAATTCAATAAAG

CAAAGTGTGCAACGCAAATGGCACCAGATTCAACTTTCAAGATCGCATTATCACTTATGGCATTTGATGCGGA

AATAATAGATCAGAAAACCATATTCAAATGGGATAAAACCCCCAAAGGAATGGAGATCTGGAACAGCAATCA

TACACCAAAGACGTGGATGCAATTTTCTGTTGTTTGGGTTTCGCAAGAAATAACCCAAAAAATTGGATTAAAT

AAAATCAAGAATTATCTCAAAGATTTTGATTATGGAAATCAAGACTTCTCTGGAGATAAAGAAAGAAACAACG

GATTAACAGAAGCATGGCTCGAAAGTAGCTTAAAAATTTCACCAGAAGAACAAATTCAATTCCTGCGTAAAAT

TATTAATCACAATCTCCCAGTTAAAAACTCAGCCATAGAAAACACCATAGAGAACATGTATCTACAAGATCTGG

ATAATAGTACAAAACTGTATGGGAAAACTGGTGCAGGATTCACAGCAAATAGAACCTTACAAAACGGATGGT

TTGAAGGGTTTATTATAAGCAAATCAGGACATAAATATGTTTTTGTGTCCGCACTTACAGGAAACTTGGGGTC

GAATTTAACATCAAGCATAAAAGCCAAGAAAAATGCGATCACCATTCTAAACACACTAAATTTATAAAAAATCT

AATGGCAAAATCGCCCAACCCTTCAATCAAGTCGGGACGGCCAAAAGCAAGCTTTTGGCTCCCCTCGCTGGCG

CTCGGCGCCCCTTATTTCAAACGTTAGACGGCAAAGTCACAGACCGCGGGATCTCTTATGACCAACTACTTTGA

TAGCCCCTTCAAAGGCAAGCTGCTTTCTGAGCAAGTGAAGAACCCCAATATCAAAGTTGGGCGGTACAGCTAT

TACTCTGGCTACTATCATGGGCACTCATTCGATGACTGCGCACGGTATCTGTTTCCGGACCGTGATGACGTTGA

TAAGTTGATCATCGGTAGTTTCTGCTCTATCGGGAGTGGGGCTTCCTTTATCATGGCTGGCAATCAGGGGCAT

CGGTACGACTGGGCATCATCTTTCCCGTTCTTTTATATGCAGGAAGAACCTGCATTCTCAAGCGCACTCGATGC

CTTCCAAAAAGCAGGTAATACTGTCATTGGCAATGACGTTTGGATCGGCTCTGAGGCAATGGTCATGCCCGGA

ATCAAGATCGGGCACGGTGCGGTGATAGGCAGCCGCTCGTTGGTGACAAAAGATGTGGGGCACTGTTGCAA

AGTTAGCGATGAGGCAGCCTTTTGTCTTATTCAAAGGCCTTACATTTCAAAAACTCTGCTTACCAGGCGCATTT

CGCCCAGGGGATCACCATAATAAAATGCTGAGGCCTGGCCTTTGCGTAGTGCACGCATCACCTCAATACCTTT

GATGGTGGCGTAAGCCGTCTTCATGGATTTAAATCCCAGCGTGGCGCCGATTATCCGTTTCAGTTTGCCATGAT

CGCATTCAATCACGTTGTTCCGGTACTTAATCTGTCGGTGTTCAACGTCAGACGGGCACCGGCCTTCGCGTTTG

AGCAGAGCAAGCGCGCGACCATAGGCGGGCGCTTTATCCGTGTTGATGAATCGCGGGATCTGCCACTTCTTCA

CGTTGTTGAGGATTTTACCCAGAAACCGGTATGCAGCTTTGCTGTTACGACGGGAGGAGAGATAAAAATCGA

CAGTGCGGCCCCGGCTGTCGACGGCCCGGTACAGATACGCCCAGCGGCCATTGACCTTCACGTAGGTTTCATC

CATGTGCCACGGGCAAAGATCGGAAGGGTTACGCCAGTACCAGCGCAGCCGTTTTTCCATTTCAGGCGCATAA

CGCTGAACCCAGCGGTAAATCGTGGAGTGATCGACATTCACTCCGCGTTCAGCCAGCATCTCCTGCAGCTCAC

GGTAACTGATGCCGTATTTGCAGTACCAGCGTACGGCCCACAGAATGATGTCACGCTGAAAATGCCGGCCTTT

GAATGGGTTCATGTGCAGCTCCATCAGCAAAAGGGGATGATAAGTTTATCACCACCGACTATTTGCAACAGTG

CCTCTCGATAGGTATAGTGTTTTGCAGTTTAGAGGAGATATCGCGATGCATACGCGGAAGGCAATAACGGAG

GCAATTCGAAAATTAGGAGTCCAAACCGGTGACCTGTTGATGGTGCATGCCTCACTTAAAGCGATTGGTCCGG

TCGAAGGAGGAGCGGAGACGGTCGTTGCCGCGTTACGCTCCGCGGTTGGGCCGACTGGCACTGTGATGGGA

TACGCGTCGTGGGACCGATCACCCTACGAGGAGACTCTGAATGGCGCTCGGTTGGATGACAAAGCCCGCCGT

ACCTGGCCGCCGTTCGATCCCGCAACGGCCGGGACTTACCGTGGGTTCGGCCTGCTGAATCAATTTCTGGTTC

AAGCCCCCGGCGCGCGGCGCAGCGCGCACCCCGATGCATCGATGGTCGCGGTTGGTCCGCTAGCTGAAACGC

TGACGGAGCCTCACGAACTCGGTCACGCCTTGGGGGAAGGGTCGCCCGTCGAGCGGTTCGTCCGCCTTGGCG

GGAAGGCCCTGCTGTTGGGTGCGCCGCTAAACTCCGTTACCGCATTGCACTACGCCGAGGCGGTTGCGGATA

TCCCCAACAAACGATGGGTGACGTATGAGATGCCGATGCTTGGAAGAAACGGTGAAGTCGCCTGGAAAACG

GCATCAGAATACGATTCAAACGGCATTCTCGATTGCTTTGCTATCGAAGGAAAGCCGGATGCGGTCGAAACTA

TAGCAAATGCTTACGTGAAGCTCGGTCGCCATCGAGAAGGTGTCGTGGGCTTTGCTCAGTGCTACCTGTTCGA

CGCGCAGGACATCGTGACGTTCGGCGTCACCTATCTTGAGAAGCACTTCGGAGCCACTCCGATCGTGCCAGCA

CACGAAGCCGCCCAGCGCTCTTGCGAGCCTTCCGGTTAGAGGCCGTCGACAATGATAATCTGGATCAACGGA

CCTTTCGGCGCCGGAAAGACGACTCTCGCTGAGCGGCTGCGCGATCGGCGTCCGAAATCGCTGATCTTTGACC

CTGAGGAAATCGGGTTCGTGGTGAAAGAAACGGTCCCCATACCAGCGAGCGGAGACTATCAGGATCTCCCCT

TGTGGAGGGGACTTACGATCGCTGCGGTCAGCGAGATTCGCAGGAATTACTCGCAGGACATCATCATCCCAA

TGACGCTCGTGCACCCGGACTATCTGACTGAGATACTCGACGGGTTAAGGCAGATCGACGATCAGCTGCTGC

ACATCTTTCTGATGCTTAACGAGGACCTATTGCGTCACCGGATCTCGAACCAGACCATGCATCCTGACCCGAAT

CGGAATGCGGAGATTCGAGAGTGGCGATTAGCGAATGTCGCCCGATGCTTGGCCGCAAGGGAACGGCTTCC

GTCCACAACCCGTGTTCTCGATAGTGGTGCACACACCAGCGATGAACTTGCAGCGATGGTGCTCGACAGACTC

GATCAGCGCACCTGATCGCCTTAGAGCTGGCTCGGGAAATCTGCACAGCGGGGATAAGTGGAGTTTCTGCCT

GACTTCGGCTTAGATGCCGGGAACAGGAGAGACCATGACGAGACGACCGCGCCGGAACCATAGCCCGGCTTT

CAAGGCGAAGGTGGCACTTGCCGCCATCCGAGGTGAGCAGACGCTGGTGGAGTTGTCCCAGCAGTTCGATGT

GCACGCCAACCAGATCAAGCAATGGAAAGACCAGCTCCTTGAGGGGGCGACAGGTGTGTTCGGCGATGAAA

CGAAAGCGGAGCCGTCGGGTCCGACCATCGATGTCAAAACGCTGCACGCGAAAATCGGCGAACTGACACTG

GAGAACGATTTTTTATCCGGTGCGCTCGGCAAGGCGGGATTGCTGGGCGGAAAGAAATGATCGACCGCGAG

CACAAGCTATCCGTCGTGCGCCAGGCGAAGCTTCTCGGCTTCAGCCGTGGCAGCGTCTACTATCTGCCTCGTC

CGGTGTCTGACGGCGATCTGGCCCTTATGCGCCGGATTGACGAATTGCATCTCGACTACCCCTTTGCCGGAAG

TCGGATGTTGCAAGGGCTCTTGAGAGGAGAAGGTCTGGAGACCGGGCGGCGACACGTCGCCACGCTGATGA

AGAAGATGGGCATCAAGGCGATCTACCGCCGCCCGAACACCTCGAAACCAGCGCCAGGGCACATACGAAAGC

TGGCAGTCACCCGGCCCAATCAGGTGTGGGCAATGGACCTGACCTACATCCCCATGGCGCGGGGATTTGTCTA

TCTGTGCGCCGTCGTGGACTGGTTCAGCCGGAAGGTCTTGTCATGGCGGTTGTCGATCACGATGGAAGCAGC

CTTCTGCATCGAGGCGGTGGAGGAGGCACTTGCCCGTCATGGCAGGCCGGAAATCTTCAATACCGACCAGGG

ATCGCAGTTCACCTCCATCGACTTCACCGCCGTGCTGAAGAGGTCACAGATTGCCATCTCGATGGATGGCAAG

GGTGCGTGGCGAGACAATGTCTTCGTCGAGCGGCTCTGGCGTTCGATCAAATACGAGGAAGTCTACCTCCAT

GCCTACAAGACTGTGTCCGAGGCACGCGCTGGCATCGCCCGATATCTGAACTTCTACAACACCAGACGCCCAC

ATTGGCACTGTTGCAAAGTTAGCGATGAGGCAGCCTTTTGTCTTATTCAAAGGCCTTACATTTCAAAAACTCTG

CTTACCAGGCGCATTTCGCCCAGGGGATCACCATAATAAAATGCTGAGGCCTGGCCTTTGCGTAGTGCACGCA

TCACCTCAATACCTTTGATGGTGGCGTAAGCCGTCTTCATGGATTTAAATCCCAGCGTGGCGCCGATTATCCGT

TTCAGTTTGCCATGATCGCATTCAATCACGTTGTTCCGGTACTTAATCTGTCGGTGTTCAACGTCAGACGGGCA

CCGGCCTTCGCGTTTGAGCAGAGCAAGCGCGACCATAGGCGGGCGCTTTATCCGTGTTGATGAATCGCGG

GATCTGCCACTTCTTCACGTTGTTGAGGATTTTACCCAGAAACCGGTATGCAGCTTTGCTGTTACGACGGGAG

GAGAGATAAAAATCGACAGTGCGGCCCCGGCTGTCGACGGCCCGGTACAGATACGCCCAGCGGCCATTGACC

TTCACGTAGGTTTCATCCATGTGCCACGGGCAAAGATCGGAAGGGTTACGCCAGTACCAGCGCAGCCGTTTTT

CCATTTCAGGCGCATAACGCTGAACCCAGCGGTAAATCGTGGAGTGATCGACATTCACTCCGCGTTCAGCCAG

CATCTCCTGCAGCTCACGGTAACTGATGCCGTATTTGCAGTACCAGCGTACGGCCCACAGAATGATGTCACGC

TGAAAATGCCGGCCTTTGAATGGGTTCATGTGCAGCTCCATCAGCAAAAGGGGATGATAAGTTTATCACCACC

GACTATTTGCAACAGTGCCCAAATGGTGGTCACCATCCTGTCGGCTGTGGCACAGGCTGAACGCCGGAGGAT

CCTAGAACGCACGAATGAGGGCCGACAGGAAGCAAAGCTGAAAGGAATCAAATTTGGCCGCAGGCGTACCG

TGGACAGGAACGTCGTGCTGACGCTTCATCAGAAGGGCACTGGTGCAACGGAAATTGCTCATCAGCTCAGTA

TTGCCCGCTCCACGGTTTATAAAATTCTTGAAGACGAAAGGGCCTCGTGATACGCCTATTTTTATAGGTTAATG

TCATGATAATAATGGTTTCTTAGACGTCAGGTGGCACTTTTCGGGGAAATGTGCGCGGAACCCCTATTTGTTTA

TTTTTCTAAATACATTCAAATATGTATCCGCTCATGAGACAATAACCCTGGTAAATGCTTCAATAATATTGAAAA

AGGAAGAGTATGAGTATTCAACATTTCGTGTCGCCCTTATTCCCTTTTTTGCGGCATTTTGCCTTCCTGTTTTTG

CTCACCCAGAAACGCTGGTGAAAGTAAAAGATGCTGAAGATCAGTTGGGTGCACGAGTGGGTTACATCGAAC

TGGATCTCAACAGCGGTAAGATCCTTGAGAGTTTTCGCCCCGAAGAACGTTTTCCAATGATGAGCACTTTTAAA

GTTCTGCTATGTGGTGCGGTATTATCCCGTGTTGACGCCGGGCAAGAGCAACTCGGTCGCCGCATACACTATT

CTCAGAATGACTTGGTTGAGTACTCACCAGTCACAGAAAAGCATCTTACGGATGGCATGACAGTAAGAGAATT

ATGCAGTGCTGCCATAACCATGAGTGATAACACTGCTGCCAACTTACTTCTGACAACGATCGGAGGACCGAAG

GAGCTAACCGCTTTTTTGCACAACATGGGGGATCATGTAACTCGCCTTGATCGTTGGGAACCGGAGCTGAATG

AAGCCATACCAAACGACGAGCGTGACACCACGATGCCTGCAGCAATGGCAACAACGTTGCGCAAACTATTAA

CTGGCGAACTACTTACTCTAGCTTCCCGGCAACAATTAATAGACTGGATGGAGGCGGATAAAGTTGCAGGACC

ACTTCTGCGCTCGGCCCTTCCGGCTGGCTGGTTTATTGCTGATAAATCTGGAGCCGGTGAGCGTGGGTCTCGC

GGTATCATTGCAGCACTGGGGCCAGATGGTAAGCCCTCCCGTATCGTAGTTATCTACACGACGGGGAGTCAG

GCAACTATGGATGAACGAAATAGACAGATCGCTGAGATAGGTGCCTCACTGATTAAGCATTGGTAACTGTCA

GACCAAGTTTACTCATATATACTTTAGATTGATTTAAAACTTCATTTTTAATTTAAAAGGATCTAGGTGAAGATC

CTTTTTGATAATCTCATGACCAAAATCCCTTAACGTGAGTTTTCGTTCCACTGAGCGTCAGACCCCGTTTCACAC

TGGTCACGCCCTCACCGACCTCAACCACCACCCCGGCACCTTCATGGCCGAGCACCACCGGGAACAGACCTTC

CGGATCATCACCGGAAAGCGTGTAGGCATCGGTATGGCATACCCCGGTGTGACTGATTTTCACCAGCACTTCA

CCTTTCTTCGGCGGTGCCACATCAATTTCAACAATTTCAAGGGGTAAACCCGGCCCGAAGGCGACTGCTGCGC

GTGATTTCATAACGGTTCCTCTGTGTCTGAGGCTTATCATGGCAGGCGATAAGCACGAATGCCGTCCACCATA

CCGCACAACAGCGCTGCCTGTATAGCGCCGGTCAGCAGGTGGCCGCCACCGGTGCCGGTTTTCTGATCAGCA

GACAGATTGCCAGCAGCAGACCGTACAGTGCCGTGCCAATCAGCCACACCGTACCGTCCGCACTCTGCTGTGT

CTGACTGAAAATAAAAGCAAACAGCAGCGGGCCCGCCACGCCGGTCAGATTGGTCAGGCTGACCAGCACACC

CTGTAGTTTTCCCTGATTTGCCGCCGATGCCCCGGCAGAGATAATGCCCTGCAATGCAGGCAGTGCAATTCCG

CCGCCTGCCAGCAGGATCAGGACCGGATACACCATCCATCCGGAAGTGATAGCAGACATCAGTAAAAACGCG

GTGGCATCGGCAATAAATCCGGCGAAAATAATGGTTTTCTCACTCAGCCGTTTTGCCAGCGCCCCGGCAACCA

CCGCCTGAAACAGTGCATGCATCGCCCCGAGTCCCGCCAGTGAAAAACCGACCGCCGCGCTGTCCCAGGCAA

AGCGGCTCTCCGTAAACAATACCCAGACAGTGGCCGGGATCTGCCCGATAAGCTGCGCGGTAAAAAGACAAA

CAGCAACAGCGCCAGAGGTTTAAGCAGTGTGATAAAGCTGATACCTGCGCTTTCTTGTTTCTGCTCCGCCGGT

TTTTCTTCTGTCTGTACCGCCGGTTTAAAGATAAAAAGACCATCAGAAAGGTGCAGGCATTCAGTATTGCCGCA

ATGACAAACGGCAGATGCGGTGAGATATCCCCCGCCAGTCCGCCGATAGCCGGCCCGGCAATCAGCCCGGCA

CCAAAGGCCGCACCGAGACGGCCGAACCAGGCGGTACGCTCGCTGACCGCCGTGCTGTCCGCCACTACCGAA

GCCGCAACCGCGCCGGTGGCACCAGTGATCCCGGAGATAATCCGCCCGAGATACAACATCCACAGCACATTG

GACAGTGCCAGCAGTGTGTAATCAAACGCGGCACCCGCCAGGGATAACAGCAGCACCGGTCTGCGCCCCAGC

TTATCTGACCATCTGCCCAGCAGCGGAGCAAAACAGACCTGCATCACCGCATACAGCGCCAGCAGAATGCCGT

AATGGTTTGCCACATCCGCTTCCGGGAGATATTCCCGCAGCAGTGACGGTAATACCGGCATGATCAGACCAAT

TCCCATCGCGTCCAGCAGTGTAATCACCAGCGCGATGACAGCGGGTTTATTCATTTTTCAGATCCTGTGTTAAT

TCCCTATCATTGATAGAGTTGGAATATATCCCTATCAATGATAGAGTGTCAATCTGAAAATCGGAGGAGACGG

TCATGGCACGGCTGAACAGAGAATCGGTTATTGATGCGGCACTGGAACTGCTGAATGAGACAGGGATTGACG

GGCTGACGACCCGCAAGCTGGCGCAGAAGCTGGGAATAGAACAGCCGACACTTTACTGGCATGTGAAAAATA

AACGGGCGTTACTGGATGCGCTGGCGGTGGAGATCCTGGCGCGTCATCATGATTATTCACTGCCTGCGGCGG

GGGAATCCTGGCAGTCATTTCTGCGCAATAATGCAATGAGTTTCCGCCGGGCGCTGCTGCGTTACCGTGACGG

GGCAAAAGTGCACCTCGGCACCCGCCCTGATGAAAAACAGTATGATACGGTGGAAACCCAGTTACGCTTTAT

GACAGAAAACGGCTTTTCACTGCGCGACGGGTTATATGCGATTTCAGCGGTCAGTCATTTTACCCTTGGTGCC

GTACTGGAGCAGCAGGAGCATACTGCCGCCCTGACCGACCGCCCTGCAGCACCGGACGAAAACCTGCCGCCG

CTATTGCGGGAAGCGCTGCAGATTATGGACAGTGATGATGGTGAGCAGGCCTTTCTGCATGGCCTGGAGAGC

CTGATCCGGGGGTTTGAGGTGCAGCTTACGGCACTGTTGCAAATAGTCGGTGGTGATAAACTTATCATCCCCT

TTTGCTGATGGAGCTGCACATGAACCCATTCAAAGGCCGGCATTTTCAGCGTGACATCATTCTGTGGGCCGTA

CGCTGGTACTGCAAATACGGCATCAGTTACCGTGAGCTGCAGGAGATGCTGGCTGAACGCGGAGTGAATGTC

GATCACTCCACGATTTACCGCTGGGTTCAGCGTTATGCGCCTGAAATGGAAAAACGGCTGCGCTGGTACTGGC

GTAACCCTTCCGATCTTTGCCCGTGGCACATGGATGAAACCTACGTGAAGGTCAATGGCCGCTGGGCGTATCT

GTACCGGGCCGTCGACAGCCGGGGCCGCACTGTCGATTTTTATCTCTCCTCCCGTCGTAACAGCAAAGCTGCA

TACCGGTTTCTGGGTAAAATCCTCAACAACGTGAAGAAGTGGCAGATCCCGCGATTCATCAACACGGATAAAG

CGCCCGCCTATGGTCGCGCGCTTGCTCTGCTCAAACGCGAAGGCCGGTGCCCGTCTGACGTTGAACACCGACA

GATTAAGTACCGGAACAACGTGATTGAATGCGATCATGGCAAACTGAAACGGATAATCGGCGCCACGCTGGG

ATTTAAATCCATGAAGACGGCTTACGCCACCATCAAAGGTATTGAGGTGATGCGTGCACTACGCAAAGGCCA

GGCCTCAGCATTTTATTATGGTGATCCCCTGGGCGAAATGCGCCTGGTAAGCAGAGTTTTTGAAATGTAAGGC

CTTTGAATAAGACAAAAGGCTGCCTCATCGCTAACTTTGCAACAGTGCCGGTACCCCATCGATGGGGGATCCT

CTAGAGTCGACCCCTCGAGGGGCATGCAAGCTTGGCGTAATCATGGTCATAGCTGTTTCCTGTGTGAAATTGT

TATCCGCTCACAATTCCACACAACATACGAGCCGGAAGCATAAAGTGTAAAGCCTGGGGTGCCTAATGAGTG

AGCTAACTCACATTAATTGCGTTGCGCTCACTGCCCGCTTTCCAGTCGGGAAACCTGTCGTGCCAGCTGCATTA

ATGAATCGGCCAACGCGCGGGGAGAGGCGGTTTGCGTATTGAGCTTGGCACTGGGCCAAGCTGAATTTCTGC

CATTCATCCGCTTATTATCACTTATTCAGGCGTAGCAACCAGGCGTTTAAGGGCACCAATAACTGCCTTAAAAA

ATTACGCCCCGCCCTGCCACTCATCGCAGTACTGTTGTAATTCATTAAGCATTCTGCCGACATGGAAGCCATCA

CAAACGGCATGATGAACCTGAATCGCCAGCGGCATCAGCACCTTGTCGCCTTGCGTATAATATTTGCCCATGG

TGAAAACGGGGGCGAAGAAGTTGTCCATATTGGCCACGTTTAAATCAAAACTGGTGAAACTCACCCAGGGAT

TGGCTGAGACGAAAAACATATTCTCAATAAACCCTTTAGGGAAATAGGCCAGGTTTTCACCGTAACACGCCAC

ATCTTGCGAATATATGTGTAGAAACTGCCGGAAATCGTCGTGGTATTCACTCCAGAGCGATGAAAACGTTTCA

GTTTGCTCATGGAAAACGGTGTAACAAGGGTGAACACTATCCCATATCACCAGCTCACCGTCTTTCATTGCCAT

ACGAAATTCCGGATGAGCATTCATCAGGCGGGCAAGAATGTGAATAAAGGCCGGATAAAACTTGTGCTTATT

TTTCTTTACGGTCTTTAAAAAGGCCGTAATATCCAGCTGAACGGTCTGGTTATAGGTACATTGAGCAACTGACT

GAAATGCCTCAAAATGTTCTTTACGATGCCATTGGGATATATCAACGGTGGTATATCCAGTGATTTTTTTCTCCA

TTTTAGCTTCCTTAGCTCCTGAAAATCTCGATAACTCAAAAAATACGCCCGGTAGTGATCTTATTTCATTATGGT

GAAAGTTGGAACCTCTTACGTGCCGATCAACGTCTCATTTTCGCCAAAAGTTGGCCCAGGGCTTCCCGGTATCA

ACAGGGACACCAGGATTTATTTATTCTGCGAAGTGATCTTCCGTTCGACGGAGTTCCACTGAGCGTCAGACCC

CGTAGAAAAGATCAAAGGATCTTCTTGAGATCCTTTTTTTCTGCGCGTAATCTGCTGCTTGCAAACAAAAAAACC

ACCGCTACCAGCGGTGGTTTGTTTGCCGGATCAAGAGCTACCAACTCTTTTTCCGAAGGTAACTGGCTTCAGCA

GAGCGCAGATACCAAATACTGTTCTTCTAGTGTAGCCGTAGTTAGGCCACCACTTCAAGAACTCTGTAGCACC

GCCTACATACCTCGCTCTGCTAATCCTGTTACCAGTGGCTGCTGCCAGTGGCGATAAGTCGTGTCTTACCGGGT

TGGACTCAAGACGATAGTTACCGGATAAGGCGCAGCGGTCGGGCTGAACGGGGGGTTCGTGCACACAGCCC

AGCTTGGAGCGAACGACCTACACCGAACTGAGATACCTACAGCGTGAGCTATGAGAAAGCGCCACGCTTCCC

GAAGGGAGAAAGGCGGACAGGTATCCGGTAAGCGGCAGGGTCGGAACAGGAGAGCGCACGAGGGAGCTTC

CAGGGGGAAACGCCTGGTATCTTTATAGTCCTGTCGGGTTTCGCCACCTCTGACTTGAGCGTCGATTTTTGTGA

TGCTCGTCAGGGGGGCGGAGCCTATGGAAAAACGCCAGCAACGCGGCCTTTTTACGGTTCCTGGCCTTTTGCTG

GCCTTTTGCTCACATGTTCTTTCCTGCGTTATCCCCTGATTCTGTGGATAACCGTATTACCGCCTTTGAGTGAGC

TGATACCGCTCGCCGCAGCCGAACGACCGAGCGCAGCGAGTCAGTGAGCGAGGAAGCGGAAGAAGCTCATT

CGCCATTCAGGCTGCGCAACTGTTGGGAAGGGCGATCGGTGCGGGCCTCTTCGCTATTACGCCAGCTGGCGA

AAGGGGGATGTGCTGCAAGGCGATTAAGTTGGGTAACGCCAGGGTTTTCCCAGTCACGACGTTGTAAAACGA

CGGCCAGTGAATTC

Supplementary Figure 6: Sequence of pHSG396:IS*26* with the translocatable unit (TU) and tandem array of TUs in the chromosome. Consensus sequence of **A**) pHSG396:IS*26* plasmid, **B**) pHSG396:IS*26* plus translocatable unit (TU) and **C**) tandem pHSG396:IS*26* plus TU

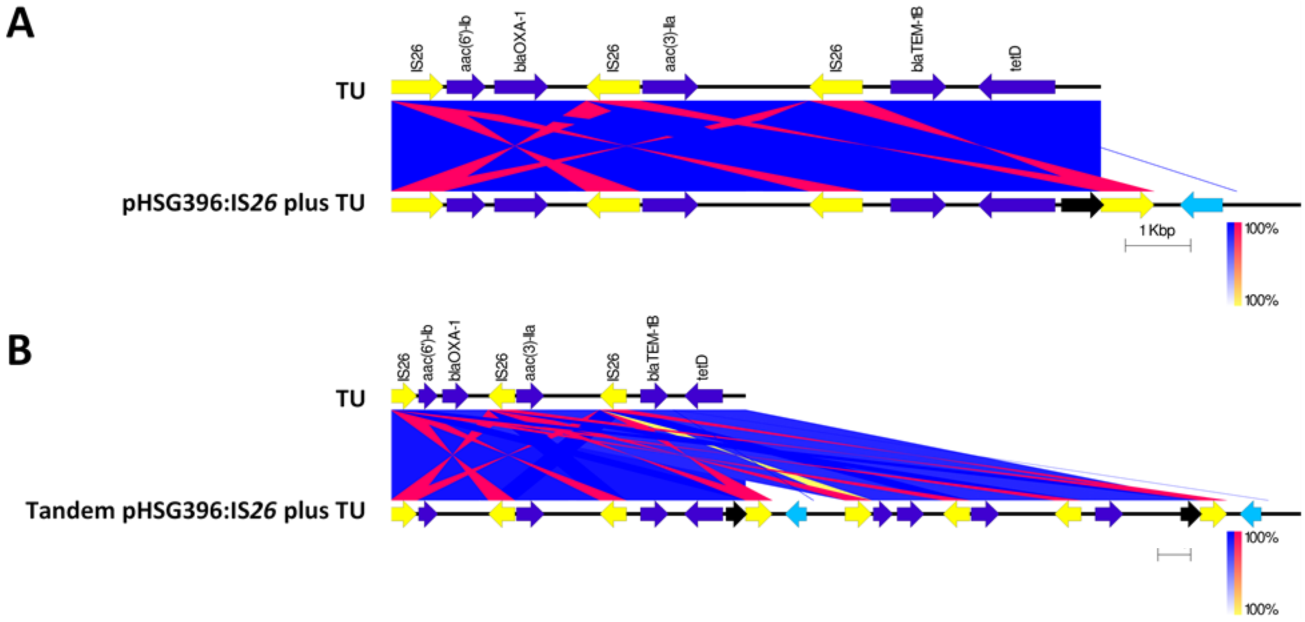

Supplementary Figure 7: The translocatable unit inserted adjacent to IS*26* in pHSG396:IS*26* following replication of the evolutionary event. Comparison of the nucleotide sequence of the translocatable unit (TU) to the consensus sequence of the predicted plasmid structures of **A**) pHSG396:IS*26* plus TU and **B**) tandem pHSG396:IS*26* plus TU. Yellow arrows represent IS*26*, blue arrows represent antimicrobial resistance genes, black arrows represent *tetR* and light blue arrows represents the chloramphenicol resistance gene of pHSG396. Percentage relatedness between the predicted and consensus sequences in the same orientation are represented in by the blue to white scale and percentage relatedness between the predicted and consensus sequences in the opposite direction are represented in the by the pink to yellow scale. Scale equals 1000 base pairs.
