# Supplementary material for: Piperacillin/tazobactam resistance in a clinical isolate of Escherichia coli due to IS26-mediated amplification of blaTEM-1B
Source: Nat Commun. 2020 Oct 1;11:4915. doi: 10.1038/s41467-020-18668-2 (PMC7530762; doi:10.1038/s41467-020-18668-2)
Supplement: Supplementary file 3 — Reporting Summary [file 41467_2020_18668_MOESM3_ESM.pdf]

nature research

Corresponding author(s): Alasdair Hubbard and Thomas Edwards

Last updated by author(s): Aug 21, 2020

Reporting Summary

Nature Research wishes to improve the reproducibility of the work that we publish. This form provides structure for consistency and transparency in reporting. For further information on Nature Research policies, see our Editorial Policies and the Editorial Policy Checklist.

Statistics

For all statistical analyses, confirm that the following items are present in the figure legend, table legend, main text, or Methods section.

n/a	Confirmed	
☐	☒	The exact sample size (*n*) for each experimental group/condition, given as a discrete number and unit of measurement
☐	☒	A statement on whether measurements were taken from distinct samples or whether the same sample was measured repeatedly
☐	☒	The statistical test(s) used AND whether they are one- or two-sided<br>*Only common tests should be described solely by name; describe more complex techniques in the Methods section.*
☒	☐	A description of all covariates tested
☒	☐	A description of any assumptions or corrections, such as tests of normality and adjustment for multiple comparisons
☐	☒	A full description of the statistical parameters including central tendency (e.g. means) or other basic estimates (e.g. regression coefficient) AND variation (e.g. standard deviation) or associated estimates of uncertainty (e.g. confidence intervals)
☐	☒	For null hypothesis testing, the test statistic (e.g. *F*, *t*, *r*) with confidence intervals, effect sizes, degrees of freedom and *P* value noted<br>*Give P values as exact values whenever suitable.*
☒	☐	For Bayesian analysis, information on the choice of priors and Markov chain Monte Carlo settings
☒	☐	For hierarchical and complex designs, identification of the appropriate level for tests and full reporting of outcomes
☒	☐	Estimates of effect sizes (e.g. Cohen's *d*, Pearson's *r*), indicating how they were calculated

Our web collection on statistics for biologists contains articles on many of the points above.

Software and code

Policy information about availability of computer code

Data collection	Oxford Nanopore Technologies sequencing reads were collected and basecalled using MinKNOW version 19.05.0<br>Relative fitness optical density was collected using Multi-user Reader Control (Clariostar Plus) version 5.61<br>qPCR ct values were collected using Rotor-Gene q series version 2.3.1<br>Nitrocefin assay optical density was collected using Multi-user Reader Control (SPECTROstar OMEGA) version 3.31
Data analysis	Oxford Nanopore Technologies sequencing were demultiplexed using Porechop version 0.2.4, quality filtered using FIltlong version 0.2.0.<br>Hybrid assembly of long and short read sequencing was performed using Unicycler version 0.4.7, assembly quality assessed using QUAST version 5.0.2, annotated using Prokka version 1.14.0 and visualized using Bandage version 0.8.1.<br>Sequence type and serotyping of the isolates were performed using MLST version 2.0.4 and SerotypeFinder version 2.0.1.<br>Genome relatedness was performed using MUMmer version 3.23 and OrthoANR version 0.93.1.<br>Antimicrobial resistance genes present in the genome were detected using ResFinder version 3.2 and plasmid replicons found using PlasmidFinder version 2.0.1<br>Long sequencing reads were mapped with BWA MEM version 0.7.17-r1188 and aligned using samtools version 1.10<br>Consensus sequences of long reads were built using Medaka version 0.11.5 and compared using EasyFig version 2.2.2<br>Genetic context of the translocatable unit and capture plasmids were performed using SnapGene version 3.3.4<br>Relative fitness was calculated using BAT version 2.1<br>All statistical analysis was performed using GraphPad Prism version 8.2.1

For manuscripts utilizing custom algorithms or software that are central to the research but not yet described in published literature, software must be made available to editors and reviewers. We strongly encourage code deposition in a community repository (e.g. GitHub). See the Nature Research guidelines for submitting code & software for further information.

Data

Policy information about availability of data

All manuscripts must include a data availability statement. This statement should provide the following information, where applicable:

- Accession codes, unique identifiers, or web links for publicly available datasets
- A list of figures that have associated raw data
- A description of any restrictions on data availability

All sequencing reads and assemblies were deposited to GenBank under the BioProject number PRJNA607545 [https://www.ncbi.nlm.nih.gov/bioproject/PRJNA607545/]. Consensus sequences of pHSG396:IS26, pHSG396:IS26 plus translocatable unit (TU) and tandem pHSG396:IS26 plus TU are available in Supplementary Figure 7.

Field-specific reporting

Please select the one below that is the best fit for your research. If you are not sure, read the appropriate sections before making your selection.

[x] Life sciences [ ] Behavioural & social sciences [ ] Ecological, evolutionary & environmental sciences

For a reference copy of the document with all sections, see nature.com/documents/nr-reporting-summary-flat.pdf

Life sciences study design

All studies must disclose on these points even when the disclosure is negative.

Sample size	The sample size was dictated by the identification of clonal Escherichia coli isolates in the clinical collection, which were isolated from the same patient and either susceptible or resistant to piperacillin/tazobactam. From the clinical isolate collection we were only able to identify one pair of clonal isolates which came from the same patient and where either susceptible or resistant to piperacillin/tazobactam which were determined by restriction fragment length polymorphism, whole genome sequencing and minimum inhibitory concentration. The mechanism of resistance was determined and reported for this clinical isolate only, therefore additional isolates or increased sample size was not required.
Data exclusions	No data were excluded
Replication	Minimum inhibitory concentrations were performed in triplicate, each with three technical replicates<br>qPCR assays were replicated in triplicate or quadruplicate<br>Nitrocefin assay were performed in triplicate<br>Comparative fitness assay were performed in triplicate, each with two technical replicates<br>All attempts at replication of the assays above were successful<br>Clinical disc diffusion testing were only performed once, which followed standard clinical practice guidelines for susceptibility testing and susceptibility to piperacillin/tazobactam was subsequently confirmed in the research laboratory<br>PCR, enzyme restriction digest and cloning were performed once and confirmed to be the correct products by sequencing, therefore replicates were not required<br>Whole genome sequencing was performed once as coverage of the genome of both clinical isolates was sufficient to achieve a good assembly of the genomes.
Randomization	Randomization was not required in this study as the bacterial isolates were specifically chosen due to their antimicrobial resistance profile, relation to other bacterial isolates in the isolate collection and whether they were isolated from a single patient to enable comparison to detect the mechanism of piperacillin/tazobactam resistance mechanism. As this information was required for selection and inclusion in the study, randomization was not relevant.
Blinding	Blinding of samples were not required in this study as the bacterial isolates were specifically chosen due to their antimicrobial resistance profile, relation to other bacterial isolates in the isolate collection and whether they were isolated from a single patient to enable comparison to detect the mechanism of piperacillin/tazobactam resistance mechanism. As this information was required for selection and inclusion in the study, blinding was not relevant.

Reporting for specific materials, systems and methods

We require information from authors about some types of materials, experimental systems and methods used in many studies. Here, indicate whether each material, system or method listed is relevant to your study. If you are not sure if a list item applies to your research, read the appropriate section before selecting a response.

Materials & experimental systems

n/a	Involved in the study
☒	☐ Antibodies
☒	☐ Eukaryotic cell lines
☒	☐ Palaeontology and archaeology
☒	☐ Animals and other organisms
☒	☐ Human research participants
☒	☐ Clinical data
☒	☐ Dual use research of concern

Methods

n/a	Involved in the study
☒	☐ ChIP-seq
☒	☐ Flow cytometry
☒	☐ MRI-based neuroimaging
